# Digital Health Interventions for Musculoskeletal Pain Conditions: Systematic Review and Meta-analysis of Randomized Controlled Trials

**DOI:** 10.2196/37869

**Published:** 2022-09-06

**Authors:** Pim Peter Valentijn, Liza Tymchenko, Teddy Jacobson, Jakob Kromann, Claus W Biermann, Mohamed Atef AlMoslemany, Rosa Ymkje Arends

**Affiliations:** 1 Essenburgh Research & Consultancy Essenburgh Group Harderwijk Netherlands; 2 Department of Health Services Research School for Public Health and Primary Care, Faculty of Health, Medicine and Life Sciences Maastricht University Maastricht Netherlands; 3 Nordic Netcare Copenhagen Denmark; 4 Faculty of Medicine Menoufia University Menoufia Egypt; 5 University of Applied Sciences Utrecht Utrecht Netherlands

**Keywords:** eHealth, models of care, mobile health, mHealth, digital health, pain, telehealth, telemedicine, disability, function, quality of life, mobile phone

## Abstract

**Background:**

Digital health solutions can provide populations with musculoskeletal pain with high-reach, low-cost, easily accessible, and scalable patient education and self-management interventions that meet the time and resource restrictions.

**Objective:**

The main objective of this study was to determine the effectiveness of digital health interventions for people with musculoskeletal pain conditions (ie, low back pain, neck pain, shoulder pain, knee pain, elbow pain, ankle pain, and whiplash).

**Methods:**

A systematic review and meta-analysis was conducted. We searched PubMed and Cochrane Central Register of Controlled Trials (from 1974 to August 2021) and selected randomized controlled trials of digital health interventions in the target population of patients with musculoskeletal pain with a minimum follow-up of 1 month. A total of 2 researchers independently screened and extracted the data.

**Results:**

A total of 56 eligible studies were included covering 9359 participants, with a mean follow-up of 25 (SD 15.48) weeks. In moderate-quality evidence, digital health interventions had a small effect on pain (standardized mean difference [SMD] 0.19, 95% CI 0.06-0.32), disability (SMD 0.14, 95% CI 0.03-0.25), quality of life (SMD 0.22, 95% CI 0.07-0.36), emotional functioning (SMD 0.24, 95% CI 0.12-0.35), and self-management (SMD 0.14, 95% CI 0.05-0.24).

**Conclusions:**

Moderate-quality evidence supports the conclusion that digital health interventions are effective in reducing pain and improving functioning and self-management of musculoskeletal pain conditions. Low-quality evidence indicates that digital health interventions can improve the quality of life and global treatment. Little research has been conducted on the influence of digital health on expenses, knowledge, overall improvement, range of motion, muscle strength, and implementation fidelity.

**Trial Registration:**

PROSPERO CRD42022307504; https://tinyurl.com/2cd25hus

## Introduction

Musculoskeletal conditions are considered the leading cause of global morbidity and have substantial individual, societal, and economic implications [1]. Musculoskeletal conditions account for one-fifth of the world’s total number of years lived with disability [1]. The burden of musculoskeletal conditions is predicted to increase dramatically in the coming years because of the aging population in Western countries. Musculoskeletal conditions include a broad range of health conditions affecting the bones, joints, muscles, and spine, as well as rare autoimmune conditions. Common symptoms include pain, stiffness, and loss of mobility and dexterity, which often interfere with people’s ability to perform daily activities. In the global research on the burden of disease, low back and neck pain were responsible for 70% of impairments [2]. The management of musculoskeletal pain conditions requires an evidence-informed innovative care model that stimulates self-management, including daily activities, self-care, patient-professional collaboration, and a collaborative practice model [3].

**Figure 1:**
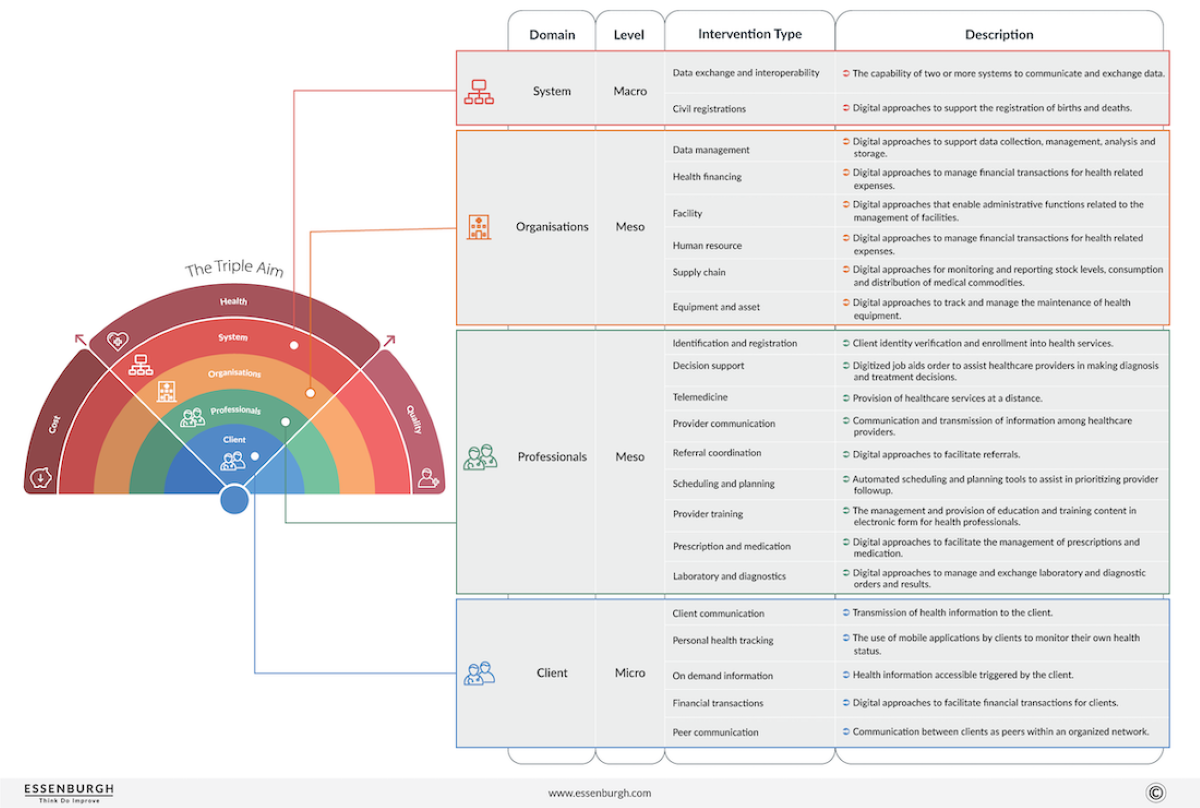
Rainbow model for digital health interventions [14].

For musculoskeletal pain conditions, there has been increasing interest in integrating digital health interventions to accomplish the triple aim of better health outcomes, better patient experiences, and smarter use of health service resources. Various studies have found moderate-quality evidence that digital health interventions have a positive clinical benefit in the management of musculoskeletal conditions leading to pain and functional disability [4-7]. However, owing to differences in content, duration, and delivery, it is difficult to draw strong conclusions about the effectiveness of digital health interventions. Hence, little is known about which type or combination of digital health solutions is superior [5,8-10]. This lack of information serves as a barrier to identifying key characteristics aligned with effective and ineffective digital health solutions and their wider implementation. Recently, the World Health Organization (WHO) published a taxonomy for the standardization of various digital health interventions and vocabulary [11]. Although taxonomy is a useful tool to differentiate between the different types of digital interventions, it cannot distinguish between the micro, meso, and macro factors that influence digital health innovation and implementation [12]. This calls for a broad overview of the evidence by outlining digital health solutions at the patient, professional, provider, and system levels, as described by the Rainbow Model (Figure 1) [13]. It is important to identify the most effective type of digital health intervention and, in turn, the most efficacious combination of components (eg, patient, provider, organizational, and system level) for clinical and managerial responses to the evidence, as well as for policy decision-making.

Following the Rainbow Model of Integrated Care (RMIC) and WHO digital health taxonomy, we comprehensively analyzed the effectiveness of digital health interventions for musculoskeletal pain conditions in published randomized controlled trials (RCTs) and assessed the extent to which differences in outcomes may be explained by the different types of interventions.

## Methods

A systematic review was conducted according to a protocol registered on PROSPERO (International Prospective Register of Systematic Reviews; registration number 307504) and the PRISMA (Preferred Reporting Items for Systematic Reviews and Meta-Analyses) guidelines [15].

### Literature Search

We searched the electronic databases PubMed and the Cochrane Library using musculoskeletal pain condition–specific and digital health–specific text words and Medical Subject Headings (Tables S1 and S2 in Multimedia Appendix 1 [16-71]) from their inception to August 2021.

### Study Selection

#### Overview

A total of 2 researchers (LT and MAA) worked separately on study selection, eligibility criteria evaluation, risk-of-bias assessment, and data extraction, and disagreements were resolved through iteration and discussion. If this failed, a third arbitrary resolution was performed by a third author (PPV). Studies were considered eligible if they were RCTs with a follow-up of ≥1 month; included participants aged >18 years with a musculoskeletal (chronic) pain condition (ie, low back pain, neck pain, shoulder pain, knee pain, elbow pain, ankle pain, or whiplash); and comprised an evaluation of a digital health intervention in the clinical, professional, organizational, or system domains of the RMIC [13]. Each intervention had to describe ≥1 digital health service according to the description of the WHO digital health taxonomy [11] (Table S3 in Multimedia Appendix 1 [16-71]). Non-English studies were excluded from this review.

#### Data Extraction and Risk-of-Bias Assessment

For each included study, 2 researchers (LT and MAA) independently extracted the data using a standardized data extraction form. Any inconsistency was resolved through iteration and discussion. When the required data were not reported in the article, the researchers contacted the authors for the missing information. If the required data could not be provided, the study was included only for qualitative review. The following methodological risks of bias were assessed for each selected study: sequence generation; allocation concealment; blinding of outcome assessors, care providers, and participants; completeness of outcome data; intention-to-treat analysis; and sponsor involvement in authorship [72]. The Covidence software was used to manage data extraction and risk-of-bias assessments [73].

#### Data Synthesis and Analysis

The primary outcomes included pain, functioning, and quality of life, as assessed using recognized and validated measures [74]. Cost, emotional functioning, overall progress, range of motion, muscle strength, knowledge, self-management, and process-related outcomes were all secondary outcomes of interest.

A 3-step method was used to identify distinct subgroups of digital health interventions according to the domains of the RMIC. First, the appropriate number of clusters was determined through a hierarchical agglomerative clustering analysis using the Euclidean distance and average silhouette methods, which measures the quality of a cluster. We tested for outliers by using the cluster membership of the distance method, which indicates how well an observation fits into the cluster that it has been assigned to [75]. No outliers were identified based on the results of this analysis. Second, a nonhierarchical cluster analysis based on the k-means algorithm was performed to validate the results of the hierarchical procedure by using the initial cluster centroid number from hierarchical clustering as a starting point [76,77]. This method establishes the presence of clusters by determining the average of all the data points in a cluster.

The grouping of the clusters was evaluated by performing a principal component analysis (PCA), which required data normalization, and the eigenvalues were calculated and analyzed in a biplot graph [78,79]. Assumptions of the PCA were tested following the procedure described by Kassambara [80] (ie, linearity of the data, level of measurement, and outliers). Finally, the clusters were visually evaluated using cluster plots and PCA. To provide an interpretation of the cluster, the cluster means of the digital health interventions were applied.

We used DerSimonian and Laird random-effects models to summarize the treatment effects and expressed the results as standardized mean differences (SMDs) for continuous outcomes using different scales together with 95% CIs. The SMD calculations were based on the effect differences between the baseline and last follow-up assessment [81]. In the systematic review, we included relevant studies; for the meta- and subgroup analyses, at least three independent studies were required to justify the meta-analysis [82].

Heterogeneity in treatment effects between studies was assessed using the restricted maximum likelihood method (*I*^2^) statistics, with *I*^2^ values of 25%, 50%, and 75% corresponding to low, moderate, and high levels of heterogeneity, respectively [83]. Potential sources of statistical heterogeneity were explored using a priori subgroup analysis to determine whether the intervention duration (1-12 months or >12 months) or setting (clinic or home-based) affected heterogeneity. Evidence of small study effects was assessed through visual examination of funnel plots [84]. We conducted a sensitivity analysis of primary outcomes by excluding studies according to the following criteria: (1) high risk of bias, (2) long follow-up (≥12 months), and (3) large sample size (>200 participants). We used a minimum of 10 independent studies [81].

Descriptive statistics were used to summarize the data, where mean and SD were reported for continuous data and frequencies and percentages for categorical data. The distribution of all continuous variables was checked. The statistical significance for subgroup and sensitivity analysis was calculated using the test for subgroup differences provided in the R Studio (version 2021.09.01) package *meta*. All analyses were performed using the statistical software R Studio (Build 372), and libraries *dmetar*, *esc*, *tidyverse*, *meta*, *grid*, *robvis*, *pvclust*, and *factorextra* were used [85].

### Quality of Evidence

The quality of evidence was rated for each pooled analysis by using the Grades of Recommendation, Assessment, Development, and Evaluation approach [86]. The quality of evidence was not downgraded for performance or detection bias as perfect blinding is considered problematic for complex digital health interventions [82]. For each comparison, 2 researchers (LT and MAA) independently rated the quality of evidence for each outcome as “high,” “moderate,” “low,” or “very low.” Discrepancies were resolved through iteration and discussion.

## Results

### Search Results and Study Characteristics

**Figure 2:**
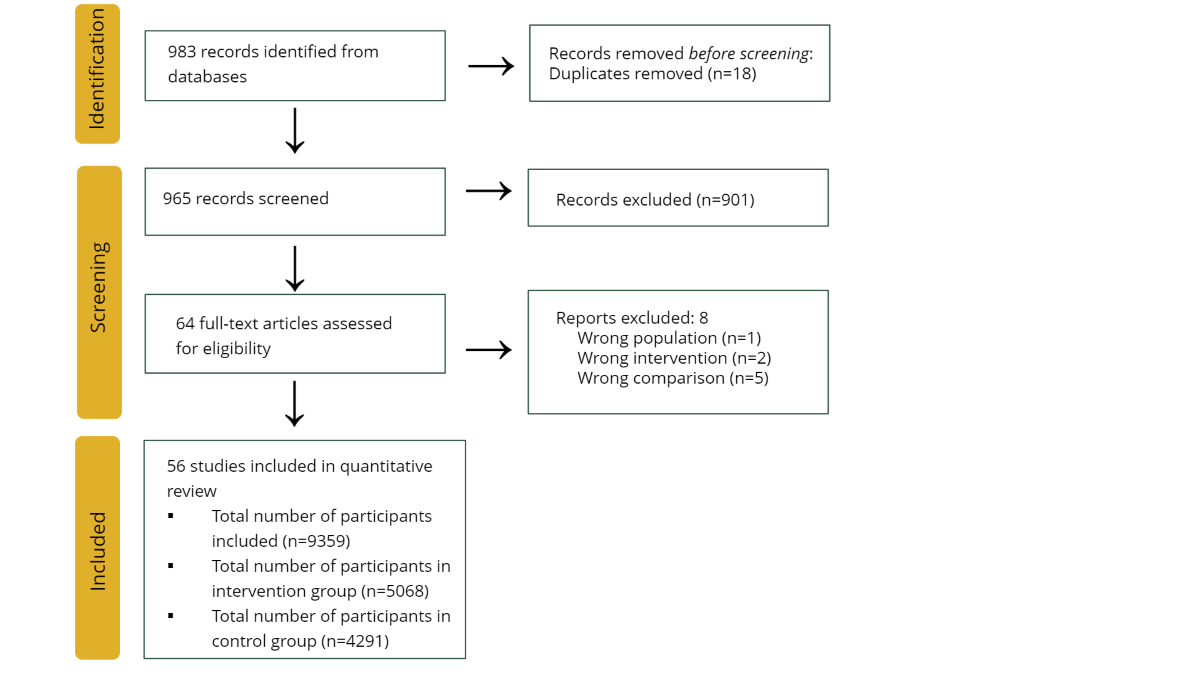
Flowchart of search strategy and study selection process.

A total of 983 publications of potential interest were identified. Of the 983 publications, after removing 18 (1.83%) duplicates, 965 (98.17%) publications were selected for title and abstract screening. Subsequently, of the 965 publications, 64 (6.63%) were selected for full-text screening, and 56 (5.8%) RCTs were considered eligible for inclusion, assessing 9359 participants. Approximately 6% (4/64) of studies reported incomplete outcomes; therefore, they were excluded from the effect analysis (Figure 2).

### Intervention Characteristics

The characteristics of the interventions in the included studies are summarized in Tables S4 and S5 in Multimedia Appendix 1. All studies performed interventions in the home base of the participants, and in some cases, the setting of the study was a combination of the home base of the participants and the primary care clinic (26/56, 46%). In addition, of the 56 studies, we included 30 (54%) studies targeting musculoskeletal pain conditions, 10 (18%) studies targeting chronic pain conditions, 9 (16%) studies targeting postsurgery rehabilitation participants, and 7 (13%) studies focusing on patients with arthritis. Most of the interventions focused on patient conditions, such as target client communication (42/56, 75% studies; 6806/9359, 72.72% participants) and personal health tracking (38/56, 68% studies; 5881/9359, 62.84% participants). Digital health interventions at the professional level included telemedicine (55/56, 98% studies; 9331/9359, 99.7% participants), client information and registration (47/56, 84% studies; 8041/9359, 85.92% participants), health care provider decision support (23/56, 41% studies; 4520/9359, 48.3% participants), health care training (23/56, 41% studies; 4569/9359, 48.82% participants), health care provider communication (12/56, 21% studies; 2901/9359, 31% participants), and referral coordination (4/56, 7% studies; 527/9359, 5.63% participants). None of the studies incorporated health care providers in a scheduled activity planning intervention. Only 9% (5/56) of studies were targeted at the organizational level. All of these studies included health financing interventions (2363/9359, 25.25% participants). Furthermore, some studies were targeted at the system level and included data collection, management, and use interventions (23/56, 41% studies; 4648/9359, 49.66% participants). The duration of the interventions ranged from 2 weeks to 12 months (median 12 weeks). Two distinctive subgroups of digital health interventions were identified in the 56 articles.

The first cluster (32/56, 57% studies; 4565/9359, 48.78% participants) included interventions mainly in professional and client domains, mostly performed (23/32, 72%) in the home base of the participant. The second cluster (24/56, 43% studies; 4794/9359, 51.22% participants) comprised interventions in the organizational, professional, and client domains, mostly performed (17/24, 71%) in the home base and clinic settings. Four statistically significant differences across the subgroups for digital health interventions were identified through the cluster differences analysis: targeted health care provider decision assistance; referral coordination; health finance; and data collection, management, and use. The 2 clusters were named based on the characteristics of their digital health interventions (Table 1).

**Table 1:** Clusters of digital health interventions (N=56).

Rainbow model intervention characterization			Total studies, n (%)	Cluster 1: patient-provider–level digital health interventions (n=32), n (%)	Cluster 2: patient-provider-organizational–level digital health interventions (n=24), n (%)	Cluster differences (*P* value)	
**Organizational domain**							
	Health financing	5 (9)		0 (0)	5 (21)	.006^a^	
	Data collection, management, and use	23 (41)		3 (9)	20 (83)	<.001^b^	
**Professional domain**							
	Client identification and registration	47 (84)		25 (78)	22 (92)	.18	
	Health care provider decision support	23 (41)		7 (22)	16 (67)	<.001^c^	
	Telemedicine	55 (98)		32 (100)	23 (96)	.25	
	Health care provider communication	12 (21)		8 (25)	4 (17)	.46	
	Referral coordination	4 (7)		0 (0)	4 (17)	.02^b^	
	Health care provider training	23 (41)		13 (41)	10 (42)	.94	
**Client domain**							
	Targeted client communication	42 (75)		26 (81)	16 (67)	.22	
	Personal health tracking	38 (68)		21 (66)	17 (71)	.69	

^a^Significant at level .01.

^b^Significant at level .001.

^c^Significant at level .05.

### Quality of Included Studies

**Figure 3:**
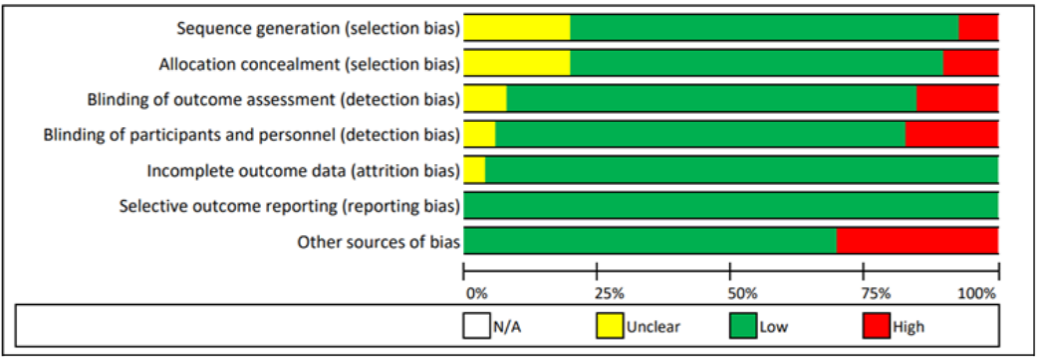
Summary of the risks of bias in included studies. For each quality item, low risk means that sufficient data were reported in the study to allow the assessment of quality, and the study fulfilled the criteria for the quality item; high risk means that sufficient data were reported in the study to assess quality, but the study did not fulfill the criteria for the quality item; and unclear risk means that incomplete data for the quality item were reported. N/A: not applicable.

The risk of bias in the included studies is summarized in Figure 3. Overall, there was a low risk of bias for 80.6% (316/392) of the items, an unclear risk for 8.7% (34/392), and a high risk for 10.7% (42/392).

### Effect of Digital Health Interventions

#### Pain

**Figure 4:**
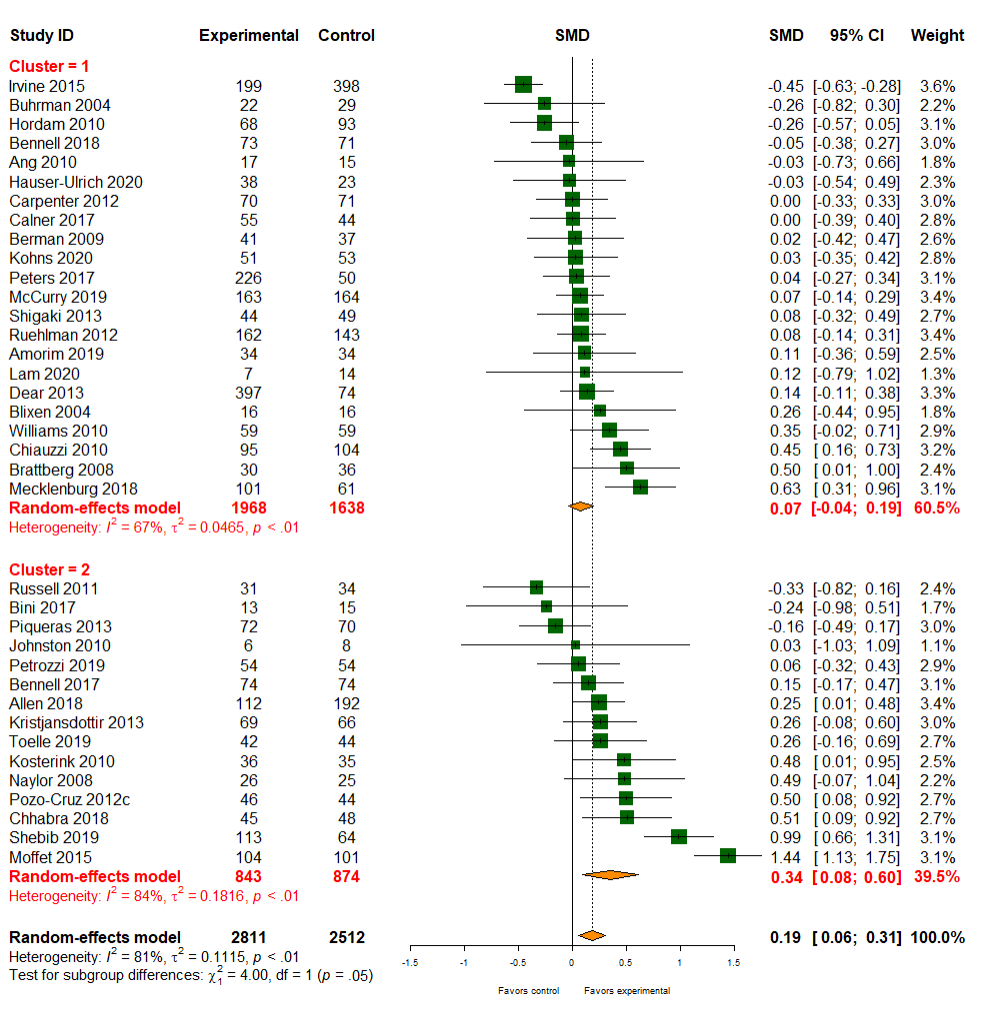
Effect of digital health on pain. SMD: standardized mean difference.

Of the 56 studies, 37 (66%; 5323/9359, 56.88% participants) reported the treatment effects on pain. Digital health interventions had a small effect on pain compared with standard care management (SMD 0.19, 95% CI 0.06-0.31; Figure 4). However, there was evidence of high heterogeneity between studies (*I*^2^=81%). There was evidence of different effects on pain based on different types of digital health interventions (patient-provider: SMD 0.07, 95% CI −0.04 to 0.19; patient-provider-organization: SMD 0.34, 95% CI 0.08-0.60; *P* value for subgroup difference=.05). The quality of the evidence for pain was rated as moderate (Table 2).

**Table 2:** Summary of findings and assessment of the quality of evidence for outcomes (N=56).

Outcomes	Studies, n (%)	Certainty assessment							Effect			Certainty
		Study design	Risk of bias	Inconsistency	Indirectness	Imprecision	Other considerations	Individuals (n=9359), n (%)		SMD^a^ rate (95% CI)		
Pain (follow-up: mean 25 weeks)	37 (66.1)	Randomized trials	Serious^b^	Not serious	Not serious	Not serious	None	5323 (56.9)		0.19 (0.06 to 0.31)	Moderate	
Disability and function (follow-up: mean 27 weeks)	30 (53.6)	Randomized trials	Serious^b^	Not serious	Not serious	Not serious	None	4849 (51.8)		0.14 (0.03 to 0.25)	Moderate	
Quality of life (follow-up: mean 25 weeks)	24 (42.9)	Randomized trials	Not serious	Not serious	Not serious	Not serious	None	3995 (42.5)		0.22 (0.07 to 0.36)	High	
Emotional functioning (follow-up: mean 29 weeks)	24 (42.9)	Randomized trials	Serious^b^	Serious^c^	Not serious	Not serious	None	3814 (40.8)		0.24 (0.12 to 0.35)	Low	
Self-management (follow-up: mean 26 weeks)	21 (37.5)	Randomized trials	Serious^b^	Not serious	Not serious	Not serious	None	2857 (30.5)		0.14 (0.05 to 0.24)	Moderate	
Global improvement (follow-up: mean 42 weeks)	4 (7.1)	Randomized trials	Serious^b^	Not serious	Not serious	Serious^d^	None	795 (5.5)		0.25 (−0.44 to 0.93)	Low	

^a^SMD: standardized mean difference.

^b^Most of the studies had a high frequency of other bias.

^c^Large heterogeneity between studies (*I*^2^>50%).

^d^95% CI includes the possible benefits from both control and digital health interventions.

#### Disability and Function

**Figure 5:**
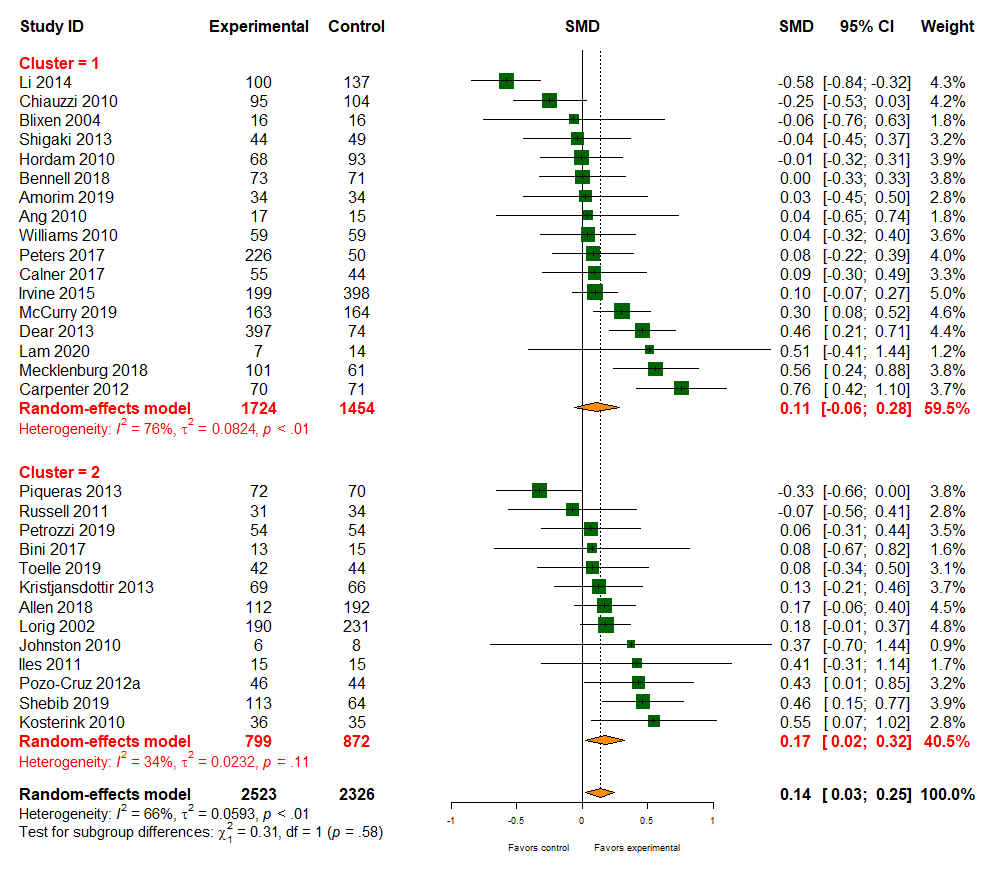
Effect of digital health on disability and function. SMD: standardized mean difference.

Of the 56 studies, data on disability and function were reported in 30 (54%) studies (4849/9359, 51.8% participants). Digital health interventions slightly improved the functioning of people with musculoskeletal conditions (SMD 0.14, 95% CI 0.03-0.25); however, there was considerable heterogeneity among studies (*I*^2^=66%; Figure 5). There was little evidence that different types of digital health interventions affected treatment effectiveness (patient-provider: SMD 0.11, 95% CI −0.06 to 0.28; patient-provider-organization: SMD 0.17, 95% CI 0.02-0.32; *P* value for subgroup difference=.58). The quality of the evidence for disability and functional outcomes was moderate.

#### Quality of Life

**Figure 6:**
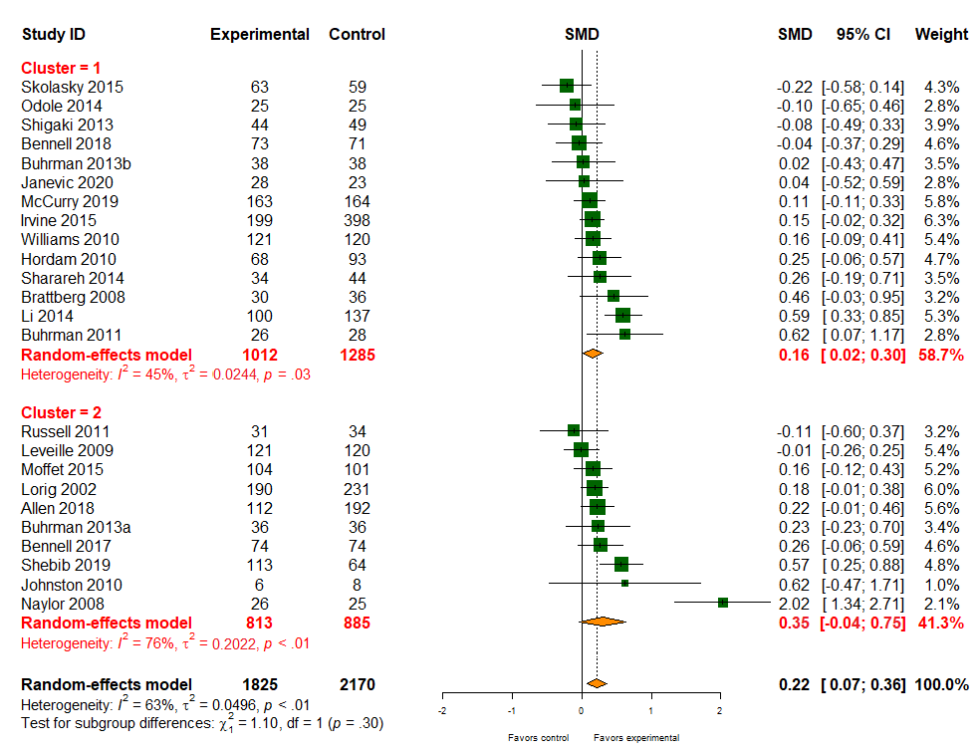
Effect of digital health on quality of life. SMD: standardized mean difference.

Digital health interventions had a slightly positive effect on health-related quality of life (24/56, 43% studies; 3995/9359, 42.69% participants; SMD 0.22, 95% CI 0.07-1.36). There was evidence of high-level heterogeneity between studies (*I*^2^=63%; Figure 6). There was little evidence that different types of digital health interventions had differing effects on quality of life (patient-provider: SMD 0.16, 95% CI 0.02-0.30; patient-provider-organization: SMD 0.35, 95% CI −0.04 to 0.75; *P* value for subgroup difference=.30). The quality of evidence for the quality of life was graded as high.

#### Emotional Functioning

**Figure 7:**
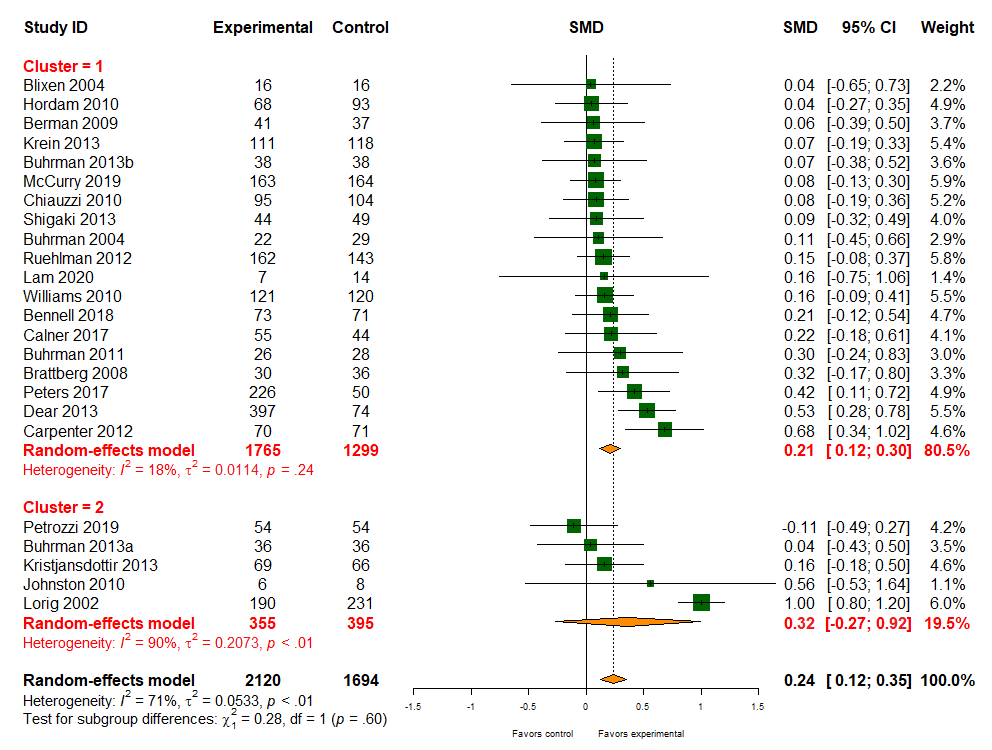
Effect of digital health on emotional functioning. SMD: standardized mean difference.

Of the 56 studies, 24 (43%; 3814/9359, 40.75% participants) reported data on emotional functioning. Digital health interventions had a positive effect on emotional functioning compared with usual care (SMD 0.24, 95% CI 0.12-0.35); however, there was evidence of heterogeneity between studies (*I*^2^=71%; Figure 7). There was little evidence of different treatment effects for different types of interventions (patient-provider: SMD 0.21, 95% CI 0.12-0.30; patient-provider-organization: SMD 0.32, 95% CI −0.27 to 0.92; *P* value for subgroup difference=.60). The quality of evidence for emotional functioning was low.

#### Self-management

**Figure 8:**
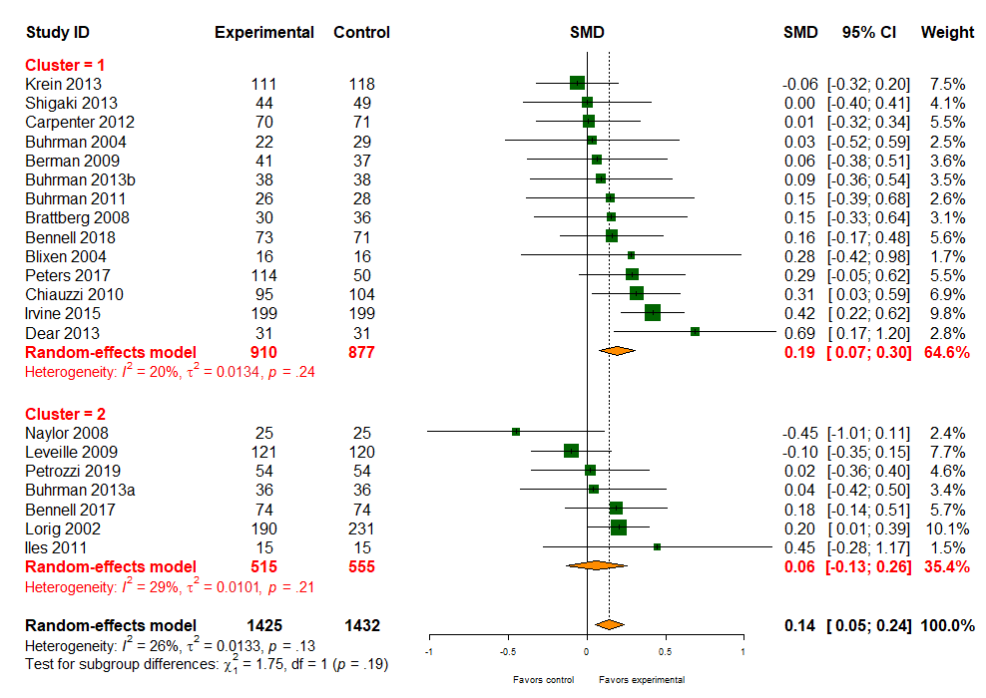
Effect of digital health on self-management. SMD: standardized mean difference.

Of the 56 studies, 21 (38%) reported treatment effects on self-management behavior (2857/9359, 30.5% participants). Evidence suggests that digital health interventions have a small positive effect on self-management behaviors compared with usual care (SMD 0.14, 95% CI 0.05-0.24; Figure 8) with moderate quality of evidence. There was little evidence that different types of interventions affected treatment effectiveness (patient-provider: SMD 0.19, 95% CI 0.07-0.30; patient-provider-organization: SMD 0.14, 95% CI −0.13 to 0.26; *P* value for subgroup difference=.19).

### Qualitative Synthesis

The qualitative analysis showed that digital health interventions have little or no effect on global improvement compared with standard care management (4/56, 7% studies, 795/9359, 8.49% participants; SMD 0.25, 95% CI −0.44 to 1.93). There was evidence of heterogeneity between studies (*I*^2^=87%), with a low quality of evidence. In addition, data on the range of motion were provided from 4% (2/56) of investigations involving 2.24% (210/9359) of participants; however, the treatment effects were highly ambiguous (Table S6 in Multimedia Appendix 1 [16-71]). Furthermore, 4% (2/56) of studies reported no effect of digital health on muscle strength (Table S6 in Multimedia Appendix 1 [16-71]). Of the 56 studies, the effects of digital health interventions on knowledge were reported in 2 (4%) studies (774/9359, 8.27% participants), and 1 (2%) study reported a significant effect (Table S6 in Multimedia Appendix 1 [16-71]). One of the studies reported an effect on satisfaction scores among participants, and another reported recovery expectation rates during the intervention (Table S6 in Multimedia Appendix 1 [16-71]). A cost analysis of digital health interventions for individuals with musculoskeletal pain conditions was presented in 4% (2/56) of studies (349/9359, 3.73% participants). In both investigations, digital health interventions were cost-effective and efficient (Table S6 in Multimedia Appendix 1 [16-71]).

### Publication Bias, Subgroup, and Sensitivity Analyses

There was little evidence of funnel plot asymmetry in treatment effects for pain, disability and function, quality of life, and emotional functioning (Figures S1-S5 in Multimedia Appendix 1 [16-71]). In addition, there was little evidence that digital health interventions had different effects on pain, disability and function, quality of life, emotional functioning, and self-management based on the duration of intervention (pain *P*=.66; disability and function *P*=.94; quality of life *P*=.45; emotional functioning *P*=.42; and self-management *P*=.66) or study setting (pain *P*=.80; disability and function *P*=.05; quality of life *P*=.63; emotional functioning *P*=.06; and self-management *P*=.06). The sensitivity analysis showed that restricting analyses to studies with lower risks of bias (pain *P*=.15; disability and function *P*=.58; quality of life *P*=.26; and self-management *P*=.39), follow-up <12 months (pain *P*=.22; disability and function *P*=.66; quality of life *P*=.31; emotional functioning *P*=.85; and self-management *P*=.48), or a small sample size (pain *P*=.88; disability and function *P*=.74; quality of life *P*=.62; emotional functioning, *P*=.19; and self-management *P*=.85) provided no different treatment effects for pain, disability and function, quality of life, and self-management (Table S7 in Multimedia Appendix 1 [16-71]). However, the risk of bias resulted in different results for emotional functioning (*P*=.01).

## Discussion

### Principal Findings

To the best of our knowledge, this meta-analytic review is the first to systematically assess the effectiveness of digital health interventions among people with musculoskeletal pain conditions. Pain, functioning, quality of life, emotional functioning, and self-management were all found to have small positive effects on a diverse set of digital health interventions. There was evidence that multicomponent interventions targeted at the client, provider, and organization levels had greater effects on pain than interventions targeted only at the client and provider levels. There was little evidence that different types of digital health interventions had different effects on other outcomes. The lack of high-quality evidence on global improvement, range of motion, muscle strength, and knowledge reinforces the need for further research on digital health for musculoskeletal pain conditions.

### Comparison With Existing Evidence

Previous reviews have also reported evidence on the effects of digital health interventions for reducing pain in musculoskeletal conditions [4,5,87-89]. However, most of these studies focused solely on chronic pain [87,88] or generic musculoskeletal conditions [5,7]. Further research is needed to corroborate our findings linking compound digital health treatments at the patient, provider, and organizational levels to reduced pain symptoms.

Our findings highlighting that digital health interventions improve function are consistent with earlier reviews of studies involving patients with generic musculoskeletal conditions [5,7]. Reviews focusing on chronic and nonspecific low back pain populations have reported limited evidence on the effects of digital health interventions on improving function [4,88]. The complexity of (chronic) pain management and the small number of RCTs included in earlier evaluations could explain the disparity in results.

This review indicates that digital health interventions have little effect on health-related quality of life. Previous systematic reviews have been inconsistent in this regard. For instance, 2 reviews suggested nonsignificant quality of life effects on musculoskeletal and chronic pain conditions [7,87], whereas 1 review reported a significant improvement in quality of life among people with nonspecific low back pain [4]. The variability of results may be explained by the differences in target populations, quality of the study design, and number of RCTs included in previous studies.

Similarly, this study has shown favorable outcomes for the emotional functioning of digital health interventions for people with musculoskeletal pain [87,88]. However, the sensitivity analysis provides evidence of the risk of bias confounding the effects, which requires further investigation. In line with other studies, this review found that digital health interventions may increase self-management behavior [88].

In all the reviewed studies, there was only a minimal reference to the cost-effectiveness of digital health interventions for musculoskeletal pain conditions. We could only include 2 studies reporting a significant cost reduction of digital health interventions compared with usual care [16,17]. Future trials should further explore whether digital health interventions can improve health outcomes related to musculoskeletal pain at lower costs than usual care. Data reporting for global improvement, range of motion, muscle strength, knowledge, and the delivery process of digital health were notably underreported, as has been observed in other reviews [4,5,87-89].

### Strengths and Limitations

This is the first review to synthesize the types of digital health interventions reported in the literature and quantify their effectiveness and confidence in treatment effects across a broad range of outcome measures. The strength of this review is that it was theoretically grounded in the WHO taxonomy [11] and the RMIC [13] to classify ambiguous digital health interventions reported in the literature. However, some limitations of this study must be acknowledged. First, it must be noted that confounding factors carry an inherent risk of bias, as evidenced by the large statistical heterogeneity across the pooled results for pain, function, quality of life, and self-management. In addition, the effects found in this study could have been influenced by differences in measurement scales and not by real differences in variability among study populations [90,91]. This should be further investigated in future studies. Moreover, the content of digital health interventions, diagnostic groups, and control conditions varied considerably, potentially biasing the results. Therefore, generalizing the overall findings to the management of musculoskeletal pain conditions should be treated with caution. Second, although we used a broad search technique, this evaluation could have been hindered by language bias, as we only included English-language literature. This means that our search may not reflect all available digital health interventions for musculoskeletal pain conditions. Third, we did not find any evidence of publication bias. It should be noted that the Egger test could potentially be misleading when used with continuous outcome measures [92]. Finally, although we abstracted and summarized the essential components of the interventions, there was minimal information on the type and intensity of digital health interventions offered.

### Relevance for Clinical Practice and Research

A major finding was that digital health interventions targeted at the clinical, provider, and organizational levels were effective in reducing pain for musculoskeletal conditions. To date, most studies have focused on isolated digital interventions targeted at the patient-provider level, such as telemedicine or targeted client communication. Future research should focus on improving the longitudinal design and on different types of interventions, drawing on the recent WHO taxonomy and the RMIC. Our findings should encourage interest in implementing real-world evaluation designs of digital health models to improve health care delivery as digital health interventions become more prevalent. Moreover, none of the studies included in this review covered the full breadth of the triple aim of assessing health, quality of care, and cost outcomes in conjunction. This emphasizes the importance of creating a core triple-aim result set for digital health interventions, which includes a defined set of outcomes that measure user experience, intervention quality, and costs.

### Conclusions

This review provides moderate-quality evidence that digital health interventions are effective in reducing pain and improving functioning and self-management of musculoskeletal pain conditions. Low-quality evidence indicates that digital health can improve the quality of life and global treatment. Although evaluations of the effects of digital health on costs, knowledge, global improvement, range of motion, muscle strength, and implementation fidelity are limited, these findings point to the need for more primary research into the particular combination of digital interventions that health care providers could use effectively.

## Appendix

Multimedia Appendix 1 Supplementary tables and figures of search strategy and study results.

## Abbreviations

PCA: principal component analysis
PRISMA: Preferred Reporting Items for Systematic Reviews and Meta-Analyses
PROSPERO: International Prospective Register of Systematic Reviews
RCT: randomized controlled trial
RMIC: Rainbow Model of Integrated Care
SMD: standardized mean difference
WHO: World Health Organization
