# Supplementary material for: Digital Health Interventions for Musculoskeletal Pain Conditions: Systematic Review and Meta-analysis of Randomized Controlled Trials
Source: J Med Internet Res. 2022 Sep 6;24(9):e37869. doi: 10.2196/37869 (PMC9490534; doi:10.2196/37869)
Supplement: Multimedia Appendix 1 [file jmir_v24i9e37869_app1.docx]

| **Population** | "back pain"[MeSH Terms] OR ("back"[All Fields] AND "pain"[All Fields]) OR "back pain"[All Fields] OR ("neck pain"[MeSH Terms] OR ("neck"[All Fields] AND "pain"[All Fields]) OR "neck pain"[All Fields]) OR ("Spin"[All Fields] AND ("pain"[MeSH Terms] OR "pain"[All Fields])) OR ("chest pain"[MeSH Terms] OR ("chest"[All Fields] AND "pain"[All Fields]) OR "chest pain"[All Fields] OR ("thoracic"[All Fields] AND "pain"[All Fields]) OR "thoracic pain"[All Fields]) OR ("neck pain"[MeSH Terms] OR ("neck"[All Fields] AND "pain"[All Fields]) OR "neck pain"[All Fields] OR ("cervical"[All Fields] AND "pain"[All Fields]) OR "cervical pain"[All Fields]) OR ("low back pain"[MeSH Terms] OR ("low"[All Fields] AND "back"[All Fields] AND "pain"[All Fields]) OR "low back pain"[All Fields] OR ("lumbar"[All Fields] AND "pain"[All Fields]) OR "lumbar pain"[All Fields]) OR ("back pain"[MeSH Terms] OR ("back"[All Fields] AND "pain"[All Fields]) OR "back pain"[All Fields] OR ("back"[All Fields] AND "ache"[All Fields]) OR "back ache"[All Fields]) OR ("low back pain"[MeSH Terms] OR ("low"[All Fields] AND "back"[All Fields] AND "pain"[All Fields]) OR "low back pain"[All Fields]) OR (("knee"[MeSH Terms] OR "knee"[All Fields] OR "knee joint"[MeSH Terms] OR ("knee"[All Fields] AND "joint"[All Fields]) OR "knee joint"[All Fields]) AND ("pain"[MeSH Terms] OR "pain"[All Fields])) OR ("shoulder pain"[MeSH Terms] OR ("shoulder"[All Fields] AND "pain"[All Fields]) OR "shoulder pain"[All Fields]) OR (("elbow"[MeSH Terms] OR "elbow"[All Fields] OR "elbow joint"[MeSH Terms] OR ("elbow"[All Fields] AND "joint"[All Fields]) OR "elbow joint"[All Fields] OR "elbow s"[All Fields] OR "elbows"[All Fields]) AND ("pain"[MeSH Terms] OR "pain"[All Fields])) OR (("wrist"[MeSH Terms] OR "wrist"[All Fields] OR "wrist joint"[MeSH Terms] OR ("wrist"[All Fields] AND "joint"[All Fields]) OR "wrist joint"[All Fields] OR "wrists"[All Fields] OR "wrist s"[All Fields]) AND ("pain"[MeSH Terms] OR "pain"[All Fields])) OR (("hand"[MeSH Terms] OR "hand"[All Fields]) AND ("pain"[MeSH Terms] OR "pain"[All Fields])) OR (("ankle"[MeSH Terms] OR "ankle"[All Fields] OR "ankle joint"[MeSH Terms] OR ("ankle"[All Fields] AND "joint"[All Fields]) OR "ankle joint"[All Fields] OR "ankles"[All Fields] OR "ankle s"[All Fields]) AND ("pain"[MeSH Terms] OR "pain"[All Fields])) OR (("foot"[MeSH Terms] OR "foot"[All Fields]) AND ("pain"[MeSH Terms] OR "pain"[All Fields])) OR (("hip"[MeSH Terms] OR "hip"[All Fields]) AND ("pain"[MeSH Terms] OR "pain"[All Fields])) OR ("musculoskeletal system"[MeSH Terms] OR ("musculoskeletal"[All Fields] AND "system"[All Fields]) OR "musculoskeletal system"[All Fields] OR "musculoskeletal"[All Fields]) |
| --- | --- |
| **Intervention** | "smartphone"[MeSH Terms] OR "smartphone"[All Fields] OR "smartphones"[All Fields] OR "smartphone s"[All Fields] OR ("smartphone"[MeSH Terms] OR "smartphone"[All Fields] OR ("smart"[All Fields] AND "phone"[All Fields]) OR "smart phone"[All Fields]) OR ("internet"[MeSH Terms] OR "internet"[All Fields] OR "internet s"[All Fields] OR "internets"[All Fields]) OR ("software"[MeSH Terms] OR "software"[All Fields] OR "software s"[All Fields] OR "softwares"[All Fields]) OR ("mobile applications"[MeSH Terms] OR ("mobile"[All Fields] AND "applications"[All Fields]) OR "mobile applications"[All Fields] OR ("mobile"[All Fields] AND "application"[All Fields]) OR "mobile application"[All Fields]) OR ("software design"[MeSH Terms] OR ("software"[All Fields] AND "design"[All Fields]) OR "software design"[All Fields]) OR ("website"[All Fields] OR "website s"[All Fields] OR "websites"[All Fields]) OR ("Web"[All Fields] AND "site"[All Fields]) OR ("webpage"[All Fields] OR "webpages"[All Fields]) OR ("Web"[All Fields] AND "page"[All Fields]) OR (("computability"[All Fields] OR "computable"[All Fields] OR "computating"[All Fields] OR "computation"[All Fields] OR "computational"[All Fields] OR "computations"[All Fields] OR "compute"[All Fields] OR "computed"[All Fields] OR "computer s"[All Fields] OR "computers"[MeSH Terms] OR "computers"[All Fields] OR "computer"[All Fields] OR "computes"[All Fields] OR "computing"[All Fields] OR "computional"[All Fields]) AND ("based"[All Fields] OR "basing"[All Fields])) OR (("computability"[All Fields] OR "computable"[All Fields] OR "computating"[All Fields] OR "computation"[All Fields] OR "computational"[All Fields] OR "computations"[All Fields] OR "compute"[All Fields] OR "computed"[All Fields] OR "computer s"[All Fields] OR "computers"[MeSH Terms] OR "computers"[All Fields] OR "computer"[All Fields] OR "computes"[All Fields] OR "computing"[All Fields] OR "computional"[All Fields]) AND ("assistances"[All Fields] OR "assistant s"[All Fields] OR "assistants"[All Fields] OR "assisted"[All Fields] OR "assisting"[All Fields] OR "assistive"[All Fields] OR "dental assistants"[MeSH Terms] OR ("dental"[All Fields] AND "assistants"[All Fields]) OR "dental assistants"[All Fields] OR "assistant"[All Fields] OR "helping behavior"[MeSH Terms] OR ("helping"[All Fields] AND "behavior"[All Fields]) OR "helping behavior"[All Fields] OR "assist"[All Fields] OR "assistance"[All Fields] OR "assists"[All Fields])) OR ("digital"[All Fields] OR "digitalisation"[All Fields] OR "digitalised"[All Fields] OR "digitalization"[All Fields] OR "digitalize"[All Fields] OR "digitalized"[All Fields] OR "digitalizer"[All Fields] OR "digitalizing"[All Fields] OR "digitally"[All Fields] OR "digitals"[All Fields] OR "digitization"[All Fields] OR "digitizations"[All Fields] OR "digitize"[All Fields] OR "digitized"[All Fields] OR "digitizer"[All Fields] OR "digitizers"[All Fields] OR "digitizes"[All Fields] OR "digitizing"[All Fields]) OR "Online"[All Fields] OR ("australas plant pathol"[Journal] OR "app"[All Fields]) OR ("appl plant sci"[Journal] OR "apps"[All Fields]) |
| **Study design** | ((("controlled clinical trial"[Publication Type] OR "controlled clinical trials as topic"[MeSH Terms] OR "controlled clinical trial"[All Fields] OR ("randomized controlled trial"[Publication Type] OR "randomized controlled trials as topic"[MeSH Terms] OR "randomised controlled trial"[All Fields] OR "randomized controlled trial"[All Fields]) OR ("clinical trial"[Publication Type] OR "clinical trials as topic"[MeSH Terms] OR "clinical trial"[All Fields]) OR "metaanalys*"[All Fields] OR (("meta"[Journal] OR "meta"[All Fields]) AND "analys*"[All Fields]) OR "meta analys*"[All Fields] OR ("systematic review"[Publication Type] OR "systematic reviews as topic"[MeSH Terms] OR "systematic review"[All Fields]) OR "random*"[All Fields] OR "trial*"[All Fields] OR "randomis*"[All Fields] OR "randomiz*"[All Fields] OR ("random*"[All Fields] AND "ADJ2"[All Fields] AND "trial*"[All Fields]) OR ("Randomly"[All Fields] AND "ADJ2"[All Fields])) AND ("allocate"[All Fields] OR "allocated"[All Fields] OR "allocates"[All Fields] OR "allocating"[All Fields] OR "allocation"[All Fields] OR "allocational"[All Fields] OR "allocations"[All Fields] OR "allocative"[All Fields] OR "allocator"[All Fields] OR "allocators"[All Fields] OR ("assign"[All Fields] OR "assignation"[All Fields] OR "assignations"[All Fields] OR "assigned"[All Fields] OR "assigning"[All Fields] OR "assignment"[All Fields] OR "assignments"[All Fields] OR "assigns"[All Fields]))) OR ("single person"[MeSH Terms] OR ("single"[All Fields] AND "person"[All Fields]) OR "single person"[All Fields] OR "single"[All Fields] OR "singles"[All Fields] OR ("double"[All Fields] OR "doubled"[All Fields] OR "doubles"[All Fields] OR "doubling"[All Fields] OR "doublings"[All Fields]) OR ("triple"[All Fields] OR "triples"[All Fields]) OR "treble"[All Fields])) AND ("blind*"[All Fields] OR "mask*"[All Fields]) |

**Table S2.** Search strategy Central

| **Population** | "arthritis" OR "osteoarthritis" OR "low back pain" OR "hip" OR "knee" OR "ankle" OR "back" OR "shoulder" OR "neck" or "cervical" OR "elbow" OR "wrist" OR "hand" OR “pain” |
| --- | --- |
| **Intervention** | "telecare" OR "telerehab*" OR "telemed*" OR "telehealth" OR "telephone" OR "internet" OR "e-health" OR "musculo*" OR "orthop*""physiotherapy" OR "physical therapy" OR “exercise” |
| **Study design** | “clinical trials” OR “randomized clinical trials” |

**Table S3.** Description digital health interventions

| **DOMAIN** | **INTERVENTION** | **DESCRIPTION** |
| --- | --- | --- |
| **Client domain** | Personal health tracking | The use of mobile applications by clients, phone based sensors, health records, and wearables for clients to monitor their own health status. This can include wearable sensors, web-tools, and apps that allow clients to review and track their health status |
|  | Targeted client communication | Transmission of targeted health information “in which separate audience segments (often demographic categories) benefit from a shared message.” Targeted communication can also be further customized according to an individual’s specific needs, resulting in “tailored client communication,” whereby message content is matched to the needs and preferences of an individual. The communication can be unidirectional and bidirectional, but initial contact is from the health system; as opposed to on-demand information service where the client initiates the first contact to the health system. |
| **Professional domain** | Client identification and registration | Client identity verification and enrollment into health services |
|  | Healthcare provider communication | Communication and transmission of information among healthcare providers, supervisors, and health system managers. |
|  | Healthcare provider decision support | Digitized record used to capture, store, access and share health information on a client or grouping of clients. |
|  | Healthcare provider training | The management and provision of education and training content in electronic form for health professionals. In contrast to decision support, healthcare provider training does not need to be used at the point of care. |
|  | Referral coordination | Digital approaches to support communication and coordination mechanisms to facilitate referrals, both within the health sector and to other health-related sectors |
|  | Telemedicine | Provision of health-care services at a distance. The delivery of health care services, where patients and providers are separated by distance. |
| **Organizational domain** | Data collection, management, and use | Digital approaches to data collection, management, analysis, storage. This can include standalone interventions focusing exclusively on data collection and management, as well as data services to support other interventions, such as data visualization within supply chain management |
|  | Health financing | Digital approaches to manage financial transactions for health system related expenses. These digital financial transactions can be used for payments to health workforce, insurance/payers, as well as administrative management of budget and expenditures. |

**Table S4.** Description of studies included in the systematic review.

| Author | Country | Target population | Setting | Intervention level (RMIC^a^) | Intervention type (WHO^b^) | Outcome assessed |
| --- | --- | --- | --- | --- | --- | --- |
| Brattberg [38] | Sweden | Female patients with fibromyalgia | Home based | - Clinical domain - Professional domain | - Targeted client communication - Personal health tracking - Telemedicine - Client identification and registration | - SF^c^-36 - HADS^d^ - PCS^e^ - CPAQ^f^ - General Self-efficacy Scale |
| Allen et al [39] | United States | Patients with knee osteoarthritis | Home and clinic | - Clinical domain - Professional domain | - Personal health tracking - Targeted client communication - Telemedicine - Client identification and registration - Data collection, management, and use | - WOMAC^g^ - The 30-second chair stand test - TUG^h^ - A 2-min step test - Unilateral stand time - The Physical Activity Scale for the Elderly - Global Assessment of Change in pain, aching, and stiffness |
| Lorig et al [40] | United States | Patients with chronic back pain | Home based | - Clinical domain - Professional domain | - Targeted client communication - Telemedicine - Client identification and registration - Health care provider decision support - Data collection, management, and use | - Pain was measured with a VAS^i^ - Revised RDQ^j^ - Illness Intrusiveness Scale - Health distress: the amount of time one has felt worried, fearful, or frustrated about health problems - Self-care orientation - Self-efficacy |
| Buhrman et al [41] | Sweden | Patients after rehabilitation of chronic pain | Home based | - Clinical domain - Professional domain | - Personal health tracking - Targeted client communication - Telemedicine - Client identification and registration - Health care provider decision support - Health care provider training - Health care provider communication - Data collection, management, and use | - CSQ^k^ - HADS - MPI^l^ - PAIRS^m^ - QOLI^n^ - CPAQ |
| Hørdam et al [42] | Denmark | Patients after hip osteoarthritis surgery | Home and clinic | - Clinical domain - Professional domain | - Targeted client communication - Telemedicine - Client identification and registration | - SF-36 |
| Buhrman et al [43] | Sweden | Patients with chronic pain | Home based | - Clinical domain - Professional domain | - Targeted client communication - Personal health tracking - Telemedicine - Health care provider training - Health care provider communication - Client identification and registration | - CPAQ - HADS - CSQ - MPI - PAIRS - QOLI |
| Bennell et al [44] | Australia | Patients with hip osteoarthritis | Home and clinic | - Clinical domain - Professional domain | - Personal health tracking - Targeted client communication - Telemedicine - Client identification and registration - Health care provider training - Health care provider decision support - Data collection, management, and use | - Hip pain on walking (11-point NPRS^o^) - WOMAC - Global change (overall, in pain and physical function) using 7-point Likert scales (NPRS Improvement) - The Assessment of Quality of Life instrument - ASES^p^ - CSQ - PCS - DASS-21^q^ - Physical Activity Scale for the Elderly |
| Berman et al [45] | United States | ≥55 patients with chronic pain | Home based | - Clinical domain - Professional domain | - Targeted client communication - Personal health tracking - Client identification and registration - Telemedicine - Health care provider communication - Health care provider decision support | - BPI^r^-short form - PSEQ^s^ - CES-D^t^ - The 6-item State-Trait Anxiety Inventory - Pain Awareness Questionnaire |
| Buhrman et al [46] | Sweden | Patients with chronic back pain | Home based | - Clinical domain - Professional domain | - Targeted client communication - Telemedicine - Client identification and registration - Health care provider communication - Health care provider training | - CSQ - MPI - PAIRS - HADS - Pain diary and NPRS - The 10-point scale measuring treatment credibility (NPRS Credibility) |
| Peters et al [47] | The Netherlands | Patients with fibromyalgia | Home based | - Clinical domain - Professional domain | - Personal health tracking - Targeted client communication - Telemedicine - Client identification and registration | - HADS - Happiness was measured with 1 item (NPRS Happiness) - FIQ^u^ - The 12-item short form of the Self-Compassion Scale - Brief Mood Introspection Scale - Life Orientation Test–Revised - Flexible Goal Adjustment scale - PCS - Perseverative Thinking Questionnaire - Illness Coping Questionnaire |
| Chhabra et al [48] | India | Patients with chronic LBP^v^ | Home based | - Clinical domain - Professional domain | - Personal health tracking - Targeted client communication - Telemedicine - Client identification and registration - Health care provider decision support - Data collection, management, and use | - NPRS - Modified ODI^w^ - Daily physical activity where the distance is measured using an activity tracker built within the app (daily physical activity) |
| Irvine et al [49], | United States | Patients with chronic LBP | Home based | - Clinical domain - Professional domain | - Targeted client communication - Telemedicine | - BPI - Dartmouth Primary Care Cooperative scale - 4 items assessing how often in the past 2 months participants engaged in behaviors intended to help or prevent back pain - The 4-item Work Limitations Questionnaire - The 6-item Stanford Presenteeism Scale - Patient Activation Measure - 14 items assessing improvement in knowledge about back pain (NPRS Knowledge) - Scale assessing participant intentions to perform the activities (behavioral inhibition system) - Scale assessing behavioral self-efficacy (NPRS Self-efficacy) - Survey of Pain Attitudes - Tampa Scale for Kinesiophobia |
| Calner et al [50], 2017 | Sweden | Patients with musculoskeletal pain from the back, neck, and shoulders and/or a generalized pain condition | Home and clinic | - Clinical domain - Professional domain | - Targeted client communication - Telemedicine - Health care provider training - Health care provider communication - Health care provider decision support - Client identification and registration | - Work Ability Index - VAS assessing pain intensity - Pain Disability Index - SF-36 |
| Mecklenburg et al [51] | United States | Patients with chronic knee pain | Home based | - Clinical domain - Professional domain | - Personal health tracking - Targeted client communication - Telemedicine - Health care provider communication - Health care provider decision support - Client identification and registration | - KOOS^x^ - KOOS Physical Function Short form - VAS for pain and stiffness score |
| Johnston et al [52] | New Zealand | Patients with chronic pain | Home based | - Clinical domain - Professional domain | - Targeted client communication - Telemedicine - Client identification and registration - Health care provider decision support - Data collection, management, and use | - CPAQ - QOLI - Satisfaction with Life Scale - Chronic Pain Values Inventory - Short-Form McGill Pain Questionnaire - Chicago Multi-scale Depression Inventory - Beck Anxiety Inventory |
| Blixen et al [53] | United States | ≥60 patients with osteoarthritis | Home based | - Clinical domain - Professional domain | - Targeted client communication - Telemedicine | - Quality of Life Survey - ASES - AIMS-2^y^ - CES-D |
| Ang et al [54] | United States | Patients with fibromyalgia | Home and clinic | - Clinical domain - Professional domain | - Personal health tracking - Targeted client communication - Telemedicine - Client identification and registration - Health care provider training - Health care provider communication - Health care provider decision support | - FIQ - PHQ^z^-8 |
| Shigaki et al [55] | United States | Patients with rheumatoid arthritis | Home based | - Clinical domain - Professional domain | - Personal health tracking - Telemedicine - Health care provider training - Client identification and registration - Data collection, management, and use | - AIMS-2 - ASES - CES-D - Quality of Life Scale - Rapid Assessment of Disease Activity in Rheumatology - SPS - University of California, Los Angeles Loneliness Scale |
| Petrozzi et al [56] | Australia | Patients with chronic LBP | Home and clinic | - Clinical domain - Professional domain - System level | - Targeted client communication - Telemedicine - Client identification and registration - Health care provider decision support - Referral coordination - Data collection, management, and use | - PSEQ - RDQ - PCS - DASS-21 - NPRS - Work Ability Index score |
| Shebib et al [57] | United States | Patients with chronic LBP | Home based | - Clinical domain - Professional domain | - Personal health tracking - Targeted client communication - Telemedicine - Client identification and registration - Data collection, management, and use | - Korff Pain - Korff Disability - ODI - VAS pain score - VAS Impact on Daily Life score - Understanding of LBP and reduction in back surgery interest (NPRS Knowledge) |
| Toelle et al [58] | Germany | Patients with chronic LBP | Home and clinic | - Clinical domain - Professional domain | - Personal health tracking - Targeted client communication - Telemedicine - Client identification and registration - Health care provider decision support - Data collection, management, and use | - An 11-point NPRS for pain intensity - Hannover Functional Ability Questionnaire - Veterans RAND 12-Item Health Survey |
| Odole and Ojo [59] | Nigeria | Patients with knee osteoarthritis | Home and clinic | - Clinical domain - Professional domain | - Targeted client communication - Telemedicine | - WHO Quality of Life-Bref |
| Krein et al [60] | United States | Adults with chronic LBP | Home based | - Clinical domain - Professional domain | - Personal health tracking - Targeted client communication - Telemedicine - Client identification and registration | - RDQ - Medical Outcomes Study - NPRS - Walking was measured as the average number of steps per day over the past 7 days - Fear-Avoidance Beliefs Questionnaire physical activity subscale - Exercise Regularly Scale |
| Chiauzzi et al [61] | United States | Patients with chronic back pain | Home based | - Clinical domain - Professional domain | - Personal health tracking - Targeted client communication - Telemedicine - Client identification and registration | - BPI - Oswestry Disability Questionnaire - DASS-21 - Patient Global Impression of Change - Chronic Pain Coping Inventory - PCS - PSEQ - Fear-Avoidance Beliefs Questionnaire |
| Buhrman et al [62] | Sweden | Patients with chronic back pain | Home based | - Clinical domain - Professional domain | - Personal health tracking - Telemedicine - Client identification and registration - Health care provider training | - CSQ - MPI - PAIRS - HADS - QOLI |
| Bennell et al [63] | Australia | Patients with chronic knee pain | Home based | - Clinical domain - Professional domain - System level | - Personal health tracking - Targeted client communication - Health care provider decision support - Health care provider training - Telemedicine - Client identification and registration - Referral coordination | - WOMAC - The Assessment of Quality of Life - ASES - The Coping Attempts Scale - PCS - Global change (overall, pain, and functional status; NPRS Improvement) - Satisfaction with treatment using a 5-point Likert scale (NPRS Satisfaction) |
| Leveille et al [64] | United States | Patients with chronic pain | Home and clinic | - Clinical domain - Professional domain - System level | - Personal health tracking - Targeted client communication - Telemedicine - Client identification and registration - Health care provider decision support - Referral coordination - Data collection, management, and use | - Days in poor physical health, days in poor mental health, and days out of normal activity (fair or poor health) - SF-36 - NPRS Mobility - General Self-efficacy Scale |
| Williams et al [65] | United States | Patients with fibromyalgia | Home and clinic | - Clinical domain - Professional domain | - Personal health tracking - Telemedicine - Health care provider training - Client identification and registration | - SF-36 - Severity Scale of the BPI - Multidimensional Fatigue Inventory - CES-D - State-Trait Personality Inventory |
| Allen et al [66] | United States | Patients with hip or knee osteoarthritis | Home and clinic | - Clinical domain - Professional domain - Organizational domain | - Targeted client communication - Telemedicine - Client identification and registration - Health care provider training - Health care provider decision support - Health financing - Data collection, management, and use | - AIMS-2 - ASES - 10 cm pain VAS |
| Ruehlman et al [67] | United States | Patients with chronic pain | Home based | - Clinical domain - Professional domain | - Targeted client communication - Telemedicine - Client identification and registration - Health care provider training - Health care provider communication | - CES-D - Profile of Chronic Pain |
| Carpenter et al [68] | United States | Patients with chronic LBP | Home based | - Clinical domain - Professional domain | - Personal health tracking - Telemedicine - Health care provider communication - Client identification and registration | - Survey of Pain Attitudes-32 - Fear-Avoidance Beliefs Questionnaire - Negative Mood Regulation Scale - PCS - RDQ - Pain Self-efficacy Scale |
| Dear et al [69] | Australia | Patients with chronic pain | Home based | - Clinical domain - Professional domain | - Personal health tracking - Targeted client communication - Telemedicine - Health care provider training - Client identification and registration | - PHQ-9 - Generalized Anxiety Disorder-7 - RDQ - Wisconsin Brief Pain Questionnaire - PSEQ - Tampa Scale for Kinesiophobia - Pain Responses Self-Statements |
| Naylor et al [70] | United States | Patients with chronic musculoskeletal pain | Home and clinic | - Clinical domain - Professional domain | - Personal health tracking - Telemedicine - Health care provider training - Health care provider decision support - Client identification and registration - Data collection, management, and use | - Short-Form McGill Pain Questionnaire - SF-36 - Treatment Outcomes in Pain Survey Total Pain Experience scale - CSQ |
| Pozo-Cruz et al [71] | Spain | Office workers with LBP | Home and clinic | - Clinical domain - Professional domain | - Targeted client communication - Telemedicine - Health care provider training - Client identification and registration - Data collection, management, and use | - LBP-Related Fitness Test - RDQ - European Quality of Life-5 Dimensions-3 Levels |
| Kristjánsdóttir et al [72] | Norway | Women with chronic widespread pain | Home and clinic | - Clinical domain - Professional domain | - Personal health tracking - Targeted client communication - Telemedicine - Health care provider decision support - Client identification and registration - Data collection, management, and use | - PCS - CPAQ - General Health Questionnaire - The Chronic Pain Values Inventory - VAS measuring the levels of pain - FIQ - (SF-8) |
| Janevic et al [73] | United States | ≥60 patients with chronic musculoskeletal pain | Home based | - Clinical domain - Professional domain | - Personal health tracking - Targeted client communication - Telemedicine - Client identification and registration | - Adherence evaluated by the percentage of days with a valid step count having a successfully logged numerical value (number of steps) - Patient-Reported Outcomes Measurement Information System |
| McCurry et al [74] | United States | ≥60 patients with persistent insomnia and chronic osteoarthritis pain | Home based | - Clinical domain - Professional domain - Organizational domain | - Targeted client communication - Telemedicine - Health care provider decision support - Health care provider communication - Health care provider training - Client identification and registration - Data collection, management and use - Health financing | - BPI-short form - PHQ-8 - Flinders Fatigue Scale - CPAQ-8 |
| Hauser-Ulrich et al [75] | Switzerland | Patients with chronic pain | Home based | - Clinical domain - Professional domain | - Personal health tracking - Telemedicine - Client identification and registration | - BPI - Visual rating scale measuring chronic pain intensity (VAS) - Working Alliance Inventory-Short Revised |
| Kohns et al [76] | United States | Patients with fibromyalgia or LBP | Home based | - Clinical domain - . Professional domain | - Targeted client communication - Telemedicine - Client identification and registration | - BPI - Patient-Reported Outcomes Measurement Information System - 4-item Psychological Attribution Scale - Pain Stages of Change Questionnaire - Tampa Kinesiophobia Scale - PCS - Satisfaction with Life Scale |
| Fatoye et al [34] | Nigeria | Patients with nonspecific chronic LBP | Home and clinic | - Clinical domain - Professional domain | - Personal health tracking - Targeted client communication - Telemedicine - Client identification and registration | - ODI - Quality-adjusted life year - Cost-utility analysis |
| Lam et al [77] | Sweden | Patients with temporomandibular disorders | Home based | - Clinical domain - Professional domain | - Personal health tracking - Targeted client communication - Telemedicine - Health care provider training - Client identification and registration | - Graded Chronic Pain Scale - PHQ-9 - The Generalized Anxiety Disorder-7 - Perceived Stress Scale-10 - PCS |
| Amorim et al [78] | Australia | Patients with chronic LBP | Home based | - Clinical domain - Professional domain | - Personal health tracking - Targeted client communication - Client identification and registration - Health care provider decision support - Telemedicine - Health care provider training | - Pain levels were assessed with an 11-point NPRS - RDQ - International Physical Activity Questionnaire - Accelerometer (ActigraphGT3X+) - DASS-21 |
| Paganini et al [33] | Germany | Patients with chronic pain | Home based | - Clinical domain - Professional domain | - Personal health tracking - Client Identification and registration - Telemedicine - Health care provider training | - Cost-effectiveness and the cost-utility analyses - Economic evaluation estimates were the incremental cost-effectiveness and cost-utility ratio |
| Skolasky et al [79] | United States | Patients who underwent surgery for degenerative lumbar spinal stenosis | Home based | - Clinical domain - Professional domain | - Targeted client communication - Telemedicine - Data collection, management, and use | - ODI - SF-12, version 2 - Hopkins Rehabilitation Engagement Rating Scale |
| Sharareh and Schwarzkopf [80] | United States | Patients who underwent total joint arthroplasty | Home based | - Clinical domain - Professional domain | - Personal health tracking - Targeted client communication - Telemedicine - Client identification and registration | - Hip Disability and Osteoarthritis Outcome Score - KOOS - Anesthesia Society of Anesthesiologists - European Quality of Life-5 Dimensions - SF-12 - University of California, Los Angeles activity scores |
| Moffet et al [81] | Canada | Patients who underwent total knee arthroplasty | Home and clinic | - Clinical domain - Professional domain - Organizational domain | - Targeted client communication - Telemedicine - Client identification and registration - Health care provider training - Health financing - Data collection, management, and use | - WOMAC - KOOS |
| Piqueras et al [82] | Spain | Patients who underwent total knee arthroplasty | Home and clinic | - Clinical domain - Professional domain | - Personal health tracking - Telemedicine - Client identification and registration - Health care provider decision support - Data collection, management, and use | - Active knee extension and active knee flexion, measured in degrees with a goniometer - Quadriceps muscle strength, measured in kilograms with the Nicholas Manual Muscle Tester dynamometer - TUG - VAS of pain - WOMAC |
| Bini and Mahajan [83] | United States | Patients who underwent total knee arthroplasty | Home and clinic | - Clinical domain - Professional domain | - Personal health tracking - Targeted client communication - Client identification and registration - Health care provider decision support - Data collection, management, and use | - 10-point VAS for pain - Veterans RAND 12-Item Health Survey - KOOS-PS |
| Russell et al [84] | Australia | Patients who underwent total knee arthroplasty | Home and clinic | - Clinical domain - Professional domain | - Personal health tracking - Telemedicine - Client identification and registration - Health care provider decision support - Data collection, management, and use | - WOMAC - Pain intensity rated on a VAS - Physical measures included active and passive knee flexion and knee extension, quadriceps muscle strength assessed by knee extension lag during a straight-leg raise, and girth measurements at the knee |
| Li et al [85] | China | Patients with total hip replacement | Home and clinic | - Clinical domain - Professional domain | - Targeted client communication - Telemedicine - Health care provider communication - Health care provider decision support - Health care provider training | - Harris Rating Scale - Total Hip Replacement (THR) Patient Medical Behavior questionnaire |
| Tousignant et al [86] | Canada | Patients who underwent total knee arthroplasty | Home and clinic | - Clinical domain - Professional domain | - Targeted client communication - Telemedicine - Health care provider training - Health care provider decision support | - Range of motion was measured using goniometry - Balance was measured using the Berg balance scale - 30-second chair stand test - WOMAC - TUG - Tinetti test - Functional Autonomy Measurement System - SF-36 |
| Iles et al [87] | Australia | Patients with LBP | Home and clinic | - Clinical domain - Professional domain - System level | - Personal health tracking - Telemedicine - Referral coordination - Health care provider training - Data collection, management, and use | - Patient-Specific Functional Scale - Score for the single-item primary nonleisure activity - Modified ODI - Recovery expectation (NPRS Recovery Expectation) - PSEQ |
| Priebe et al [88] | Germany | Patients with LBP | Home and clinic | - Clinical domain - Professional domain - Organizational domain | - Personal health tracking - Telemedicine - Health care provider training - Client identification and registration - Health care provider communication - Health care provider decision support - Health financing - Data collection, management, and use | - Pain intensity on an 11-point NPRS - DASS-21 - Veterans RAND 12-Item Health Survey |
| Kosterink et al [89] | Belgium, Germany, and the Netherlands | Patients with neck and shoulders pain | Home and clinic | - Clinical domain - Professional domain - Organizational domain | - Person health tracking - Telemedicine - Client identification and registration - Health financing - Data collection, management, and use | - Pain intensity was assessed on a VAS - The Pain Disability Index - The main resource use outcomes were the therapist time required |
| Pozo-Cruz et al [90] | Spain | Office workers with LBP | Home and clinic | - Clinical domain - Professional domain | - Personal health tracking - Telemedicine - Client identification and registration - Health care provider training - Health care provider communication - Data collection, management, and use | - European Quality of Life-5 Dimensions-3 Levels - ODI - Keele Start Back Screening Tool |
| Pozo-Cruz et al [91] | Spain | Office workers with LBP | Home and clinic | - Clinical domain - Professional domain | - Personal health tracking - Telemedicine - Client identification and registration - Health care provider training - Health care provider communication - Data collection, management, and use | - Keele Start Back Screening Tool - RDQ - European Quality of Life-5 Dimensions-3 Levels |

^a^RMIC: Rainbow Model of Integrated Care.

^b^WHO: World Health Organization.

^c^SF: Short-Form Health Survey.

^d^HADS: Hospital and Anxiety Depression Scale.

^e^PCS: Pain Catastrophizing Scale.

^f^CPAQ: Chronic Pain Acceptance Questionnaire.

^g^WOMAC: Western Ontario and McMaster Universities Osteoarthritis Index.

^h^TUG: Timed Up and Go Test.

^i^VAS: visual analog scale.

^j^RDQ: Roland-Morris Disability Questionnaire.

^k^CSQ: Coping Strategies Questionnaire.

^l^MPI: Multidimensional Pain Inventory.

^m^PAIRS: Pain and Impairment Relationship Scale.

^n^QOLI: Quality of Life Inventory.

^o^NPRS: numerical pain rating scale.

^p^ASES: Arthritis Self-efficacy Scale.

^q^DASS-21: Depression Anxiety and Stress Scale.

^r^BPI: Brief Pain Inventory.

^s^PSEQ: Pain Self-efficacy Questionnaire.

^t^CES-D: Center for Epidemiologic Studies–Short Depression.

^u^FIQ: Fibromyalgia Impact Questionnaire.

^v^LBP: low back pain.

^w^ODI: Oswestry Disability Index.

^x^KOOS: Knee Injury and Osteoarthritis Outcome Score.

^y^AIMS-2: Arthritis Impact Measurement Scale.

^z^PHQ: Patient Health Questionnaire.

**Table S5.** Characteristics of eHealth interventions included in the systematic review and meta-analysis.

| Study | Description of the intervention group | Description of the control group |
| --- | --- | --- |
| Brattberg [38] | Web-based Emotional Freedom Techniques education and digital reporting of distress rating | Wait-list control group |
| Allen et al [39] | Internet-based exercise training | Standard physical therapy and wait-list |
| Lorig et al [40] | Email discussion group; participants also received a book and videotape about back pain | Controls received a subscription to a non–health-related magazine of their choice (no intervention) |
| Buhrman et al [41] | 8 treatment modules of the internet program about chronic pain | Control group had a web-based discussion group for chronic pain |
| Hørdam et al [42] | Standard postoperative procedure in combination with telephone support and counseling | Standard postoperative procedure |
| Buhrman et al [43] | Internet-delivered ACT^a^ comprising 7 sections | Web-based discussion group for chronic pain |
| Bennell et al [44] | Web-based education and internet-based PCST^b^, as well as a home-based exercise program from physiotherapists | Web-based education and physiotherapy for home exercises |
| Berman et al [45] | Web-based mind-body self-care techniques; web-based material included audio, visual, textual, and illustrative examples and worksheets | Wait-list control group |
| Buhrman et al [46] | Internet-based behavioral intervention with telephone support | Wait-list control group |
| Peters et al [47] | An internet-delivered positive psychology program and an internet-delivered cognitive-behavioral program | Wait-list control group |
| Chhabra et al [48] | App group received Snapcare to report daily activity goals (including back and aerobic exercises), motivating, promoting, and guiding the participants to increase their level of physical activity and exercise adherence in addition to the written prescription | Conventional group received a written prescription from a physician |
| Irvine et al [49] | Fitback multiple-visit web-based program that provides education and behavioral strategies to manage current pain and prevent future pain episodes | Alternative care in the form of emailing a list with sites with back pain information; and the control group received standard care |
| Calner et al [50] | Multimodal pain rehabilitation and the web-based behavioral change program for activity | Only multimodal pain rehabilitation |
| Mecklenburg et al [51] | The Hinge Health digital care program for chronic knee pain | 3 education pieces regarding self-care for chronic knee pain |
| Johnston et al [52] | Intervention participants read the self-help book and completed exercises from it with weekly telephone support | Wait-list; each week telephone assessments |
| Blixen et al [53] | The intervention group received 6 weekly mailings of osteoarthritis health education modules, a relaxation audio tape, and 6 weekly 45-minute follow-up telephone self-management sessions | The Control group received usual care |
| Ang et al [54] | Usual care with telephone-delivered CBT^c^ in combination with a companion workbook | Usual care comprising customary care |
| Shigaki et al [55] | RAHelp website program with education and tools for participant interaction and weekly telephone contact with the leader of the program | Wait-list control group |
| Petrozzi et al [56] | Combination of standard care (physical treatments) and access to a self-administered program, MoodGYM, comprising a combination of written information, real-life examples, and quizzes delivered within the principles of a CBT framework | Standard care (physical treatments) |
| Shebib et al [57] | Intervention group received the digital care program comprising sensor-guided exercise therapy, education articles, CBT, team discussions, activity tracking, symptom tracking, and one-on-one coaching through a dedicated app | Control group received 3 digital education articles only |
| Toelle et al [58] | Intervention group used the Kaia app, which included a multidisciplinary pain treatment program | Control group received physiotherapy plus web-based patient education on various issues of back pain |
| Odole and Ojo [59] | Telephysiotherapy group received structured telephone monitoring with self-administered, specific exercises at home | Clinic group received physiotherapist-administered specific exercises in the clinic thrice weekly |
| Krein et al [60] | The intervention group received an enhanced (uploading) pedometer and had access to a website that provided automated walking goals, feedback, motivational messages, and social support through an e-community | Control group participants also received the enhanced pedometer but did not receive the automated feedback or have access to the website |
| Chiauzzi et al [61] | Website (painACTION-Back Pain) based on CBT and self-management principles; participants needed to visit the site twice a week and follow guides | Only information about back pain |
| Buhrman et al [62] | Self-help management program administered via the internet, which included 8 modules | Wait-list control group |
| Bennell et al [63] | The intervention was delivered via the internet and included educational material, 7 videoconferencing (Microsoft Skype) sessions with a physiotherapist for home exercise, and a PCST program over 3 months for participants in metropolitan and regional settings | The control group received internet-based educational material |
| Leveille et al [64] | Intervention group received a standardized PatientSite message from the nurse e-coach | Control group received a general message through PatientSite containing URL links to US government websites with general health information |
| Williams et al [65] | Usual care plus web-Enhanced behavioral self-management program comprising 13 modules, including video lectures, text summaries, homework, and self-monitoring forms | Usual care |
| Allen et al [66] | Intervention group received usual care plus self-management education material on 10 topics, an exercise video, and monthly calls with a health educator | Attention group received usual care plus video and written education material regarding different health conditions (except arthritis); control group received only usual care |
| Ruehlman et al [67] | Access to a chronic pain management program comprising 4 learning modules, including Web-based and offline activities such as educational materials, homework, and self-monitoring exercises | Wait-list control group with treatment as usual |
| Carpenter et al [68] | Access to a web-based CBT intervention, entitled the Wellness Workbook, comprising 6 sequential chapters with therapeutic content supporting cognitive and behavioral strategies, including cognitive therapy, behavioral activation, ACT, and mindfulness-based stress reduction | Wait-list control group |
| Dear et al [69] | Online pain course comprising 5 core lessons and summaries; in addition, the group was divided into 3 groups of weekly clinical contact: regular (each week), optional (if requested), and no contact | Treatment as usual wait-list control croup |
| Naylor et al [70] | Usual care plus a therapeutic interactive voice response program comprising 4 prerecorded components and monthly personalized feedback from a CBT therapist | Treatment as usual control group |
| Pozo-Cruz et al [71], 2012 | Usual care plus a web-based multidisciplinary program that included exercise and postural education | Treatment as usual control group |
| Kristjánsdóttir et al [72] | Intervention comprised a 4-week smartphone-delivered program with written diaries and therapist feedback following an inpatient chronic pain rehabilitation program; in addition, the group had access to a noninteractive website to promote constructive self-management | Control group had only access to a noninteractive website to promote constructive self-management |
| Janevic et al [73] | Intervention group received a Fitbit Zip to count their daily steps in 3 different ways: interactive voice response calls, SMS text messages, or synchronizing the device with an app | Control group had no intervention |
| McCurry et al [74] | CBT for insomnia guided by study coaches, with bed and rising time schedule, education, sleep diary, and sessions with a coach | Education-only control groups received information about osteoarthritis |
| Hauser-Ulrich et al [75] | painSELfMAnagement as a text-based health care chatbot for psychoeducation based on CBT | Wait-list group who received motivational messages with a quotation every week unrelated to chronic pain |
| Kohns et al [76] | Pain psychology and neuroscience intervention comprising video education and self-assessment exercises | Control group received a health behavior program comprising video, exercises, and rating of their health |
| Fatoye et al [34] | Telerehabilitation-based McKenzie therapy, a mobile-based app based on the McKenzie extension protocol and back care education | Clinic-based McKenzie therapy; same exercises in the physiotherapy clinic |
| Lam et al [77] | Intervention group received a dentist-assisted internet-based multimodal pain program with 7 modules based on CBT | Control group received conventional occlusal splint therapy |
| Amorim et al [78] | Intervention group received a physical activity and sedentary behavior information booklet; furthermore, they developed an activity plan with guidance from a health coach, and the group received an activity tracker (Fitbit) and access to a mobile web app (IMPACT app) to monitor their progress | Control group received the same booklet and advice to keep an active life |
| Paganini et al [33] | The intervention is based on ACT, comprising 7 modules; 2 groups with or without guidance; participants had the option to receive daily automated SMS text messages that repeated content, reminded, and motivated participants | Wait-list control group |
| Skolasky et al [79] | Intervention group received health behavior change counseling, including strategies of motivational interviewing | Control group received usual care |
| Sharareh and Schwarzkopf [80] | Intervention group received telemedicine sessions with a physician for rehabilitation | Control group received no intervention |
| Moffet et al [81] | In-home telerehabilitation, which included video sessions between the patient and therapist | Face-to-face home visits |
| Piqueras et al [82] | Intervention group received interactive virtual telerehabilitation, receiving information to perform exercises, and the therapist could virtually follow the performance | Control group received standard rehabilitation |
| Bini and Mahajan [83] | Intervention group used an asynchronous video app on a mobile device for telerehabilitation | Control group received standard rehabilitation |
| Russell et al [84] | Intervention group received internet-based telerehabilitation through real-time interaction with a physical therapist | Control group received standard rehabilitation |
| Li et al [85] | Intervention group received individualized health education and guidance, including exercise, cautions in daily life, and regular examination | Control group received usual care |
| Tousignant et al [86] | Intervention group received telerehabilitation sessions at the patient’s home via an internet connection | Control group received usual care |
| Iles et al [87] | Intervention received usual physiotherapy care in combination with telephone coaching | Control group received usual care |
| Priebe et al [88] | Intervention group received the Rise-up program where patients did a Start Back test to indicate risk groups for chronic back pain; high-risk groups received teleconsultation with a pain specialist; the intervention group also received access to the Kaia app | Control group received usual care |
| Kosterink et al [89] | Intervention group received myofeedback-based teletreatment service MyoTel; therefore, the specialist had more information about the musculoskeletal health of patients and could better prepare for the consultation | Control group received usual care |
| Pozo-Cruz et al [90] | Usual care plus a web-based multidisciplinary program that included exercise and postural education | Treatment as usual control group |
| Pozo-Cruz et al [91] | Usual care plus a web-based multidisciplinary program that included exercise and postural education | Treatment as usual control group |

**Table S6.** Effect digital health on other outcomes

| **STUDY** | **TIME-**  **POINT (months)** | **IMPROVEMENT** | **RANGE OF MOTION** | **MUSCLE STRENGTH** | **KNOWLEDGE** | **PROCESS OF CARE** | **COSTS** |
| --- | --- | --- | --- | --- | --- | --- | --- |
| **Allen 2018** [[39]](https://paperpile.com/c/YCN5Fp/Kipj1) | 12 | For left knee global assessment of change mean difference with baseline was greater in intervention group (*M*=0.57, *95% CI= 0.13 - 1.01*) than control group (*M*=0.17, *95% CI= -0.24 - 0.59*). While there were almost no differences for the right knee (*M intervention* 0.53, *M controrl group* 0.58) | - | - | - | - | - |
| **Amorim 2019** [[78]](https://paperpile.com/c/YCN5Fp/IGwBG) | 6 | - | There were no significant between-group differences (*M=* 133.5, *95% CI* -169.6 - 426.6, *P* 0.378) between intervention and control group for objectively assessed physical activity measured with accelerometer. | - | - | - | - |
| **Bennel 2017** [[63]](https://paperpile.com/c/YCN5Fp/oQYEf) | 9 | At both time points significantly (*P* 0,015) more participants in the intervention group (43,4%) reported global improvements in comparison to control group (22,9%). | - | - | - | Intervention group (*M=*1.8, *95% CI* 1.6 - 2.0) as control group (*M=*2.2, *95% CI* 2.0 - 2.4) reported high satisfaction with treatment. | - |
| **Bennell 2018** [[44]](https://paperpile.com/c/YCN5Fp/R0v6Q) | 12 | A greater percentage of participants in the intervention group (26%) reported global improvement than the comparison group (4%) at 8 weeks. | - | - | - | - | - |
| **Chiauzzi 2010** [[61]](https://paperpile.com/c/YCN5Fp/pV3a1) | 6 | The distribution of participants revealed that greater proportion of intervention participants (56,6%) reported at least a minimal improvement in their condition in comparison to control group (33%). | - | - | - | - | - |
| **Fatoye 2020** [[34]](https://paperpile.com/c/YCN5Fp/PAzTO) | 2 | - | - | - | - | - | The findings of the study suggested that digital health intervention($61,7, *QALY gained* 0,085) for people with nonspecific chronic low back pain was cost saving in comparison to clinic based intervention ($106, *QALY gained* 0,084). |
| **Iles 2011** [[87]](https://paperpile.com/c/YCN5Fp/OEuAe) | 3 | - | - | - | - | The recovery expectation in the intervention group improved significantly more than control group (*MD=*3.4, *95% CI* 1.1 - 5.7). | - |
| **Irvine 2015** [[49]](https://paperpile.com/c/YCN5Fp/yj0UJ) | 4 | - | - | - | This study indicated that intervention (*M=*0.61, *SD=* 0.14) can effectively improve user knowledge in comparison to control group (*M=*0.51, *SD=*0.16). | *-* | - |
| **Paganini 2019** [[33]](https://paperpile.com/c/YCN5Fp/sw4oR) | 6 | - | - | - | - | - | Unguided digital health intervention revealed lower costs (*QALY* 0,266, *mean costs* €6.560) at better health outcomes in comparison to guided digital health outcomes (*QALY* 0,280, *mean costs* €6.945)  . |
| **Piqueras 2013** [[82]](https://paperpile.com/c/YCN5Fp/Afk7L) | 3 | - | The increase in active knee flexion range measured with gonimeter was similar in both intervention (*M=*18.16, *SD=*9.71) and control group (*M=*15.63, *SD=*8.82), with no significant (*P* 0,193) differences observed. | The intervention group (*M=*8.48, *SD=*4.96) achieved a significant (*P* 0,018) greater muscle quadricepts strength in comparison to control group (*M=*5.89, *SD=*5.17). There were no significant (*P* 0.349) improvements in hamstring strength between both groups. | - | - | - |
| **Russell 2011** [[84]](https://paperpile.com/c/YCN5Fp/RSsw6) | 1.5 | - | - | Nonsignificant (*P* 0.23) differences between the intervention (*M=*9.24, *SD=*7.25) and control group (*M=*5.70, *SD=*5.18) were found for muscle strength. | - | - | - |
| **Shebib 2019** [[57]](https://paperpile.com/c/YCN5Fp/baK9R) | 3 | - | - | - | Participants’ understanding of condition and treatment options was significantly (P 0.0005) higher in intervention group (*M=*2.47, *SD=*1.07) in comparison to control group (*M=*1.94, *SD=*0.871). | *-* | - |

Blank cells indicate no data available for variables.

Abbreviations: M, mean of the outcome assessment; SD, standard deviation; MD, mean difference of the outcome assessment.

**Table S7.** Sensitivity analysis

| **OUTCOME MEASURES** | **PAIN** | **DISABILITY AND FUNCTION** | **QUALITY OF LIFE** | **EMOTIONAL FUNCTIONING** | **SELF-MANAGEMENT** |
| --- | --- | --- | --- | --- | --- |
| **RISK OF BIAS** | | | | | |
| **LOW** | 0,17  [-0,16;0,51]  I^2^: 88% | 0,08  [-0,10;0,27]  I^2^: 42% | 0,11  [0,00;0,22]  I^2^: 0% | 0,10  [0,03;0,17]  I^2^: 0% | 0,06  [-0,05;0,16]  I^2^: 0% |
| **UNCLEAR** | -0,01  [-0,33;0,30]  I^2^ 82% | 0,10  [0,01;0,18]  I^2^: 0% | 0,62  [-0,78;2,01]  I^2^: 89% | 0,11  [-0,05;0,27]  I^2^: 0% | 0,11  [-1,04;1,27]  I^2^: 76% |
| **HIGH** | 0,27  [0,13;0,41]  I^2^: 55% | 0,19  [0,00;0,38]  I^2^: 79% | 0,23  [0,03;0,44]  I^2^: 64% | 0,38  [0,18;0,57]  I^2^: 77% | 0,16  [0,02;0,29]  I^2^: 22% |
| **P-value** | 0,15 | 0,58 | 0,26 | 0,01** | 0,39 |
| **FOLLOW-UP** | | | | | |
| **FOLLOW-UP <12** | 0,21  [0,06;0,35]  I^2^: 83% | 0,14  [-0,01;0,28]  I^2^: 72% | 0,25  [0,06;0,44]  I^2^: 68% | 0,23  [0,13;0,33]  I^2^: 16% | 0,16  [0,04;0,28]  I^2^: 32% |
| **FOLLOW-UP >=12** | 0,10  [-0,05;0,24]  I^2^: 0% | 0,17  [0,07;0,28]  I^2^: 0% | 0,15  [0,00;0,29]  I^2^: 0% | 0,26  [-0,16;0,68]  I^2^: 91% | 0,10  [-0,11;0,31]  I^2^: 0% |
| **P-value** | 0,22 | 0,66 | 0,31 | 0,85 | 0,48 |
| **STUDY SIZE** | | | | | |
| **STUDY SIZE <200** | 0,18  [0,06;0,30]  I^2^: 59% | 0,15  [0,02;0,28]  I^2^: 55% | 0,26  [0,00;0,52]  I^2^: 70% | 0,16  [0,03;0,66]  I^2^: 89% | 0,15  [0,05;0,25]  I^2^: 0% |
| **STUDY SIZE >200** | 0,22  [-0,32’0,75]  I^2^: 95% | 0,10  [-0,19;0,40]  I^2^: 84% | 0,19  [0,06;0,32]  I^2^: 42% | 0,16  [0,06;0,26]  I^2^: 0% | 0,13  [-0,26;0,51]  I^2^: 78% |
| **P-value** | 0,88 | 0,74 | 0,62 | 0,19 | 0,85 |

*Significant at level 0.01

Results are represented as standardized mean differences (SMD), 95% Confidence interval and Restricted Maximum Likelihood method (I^2^) statistics.

Figure S1. Funnel plot pain.


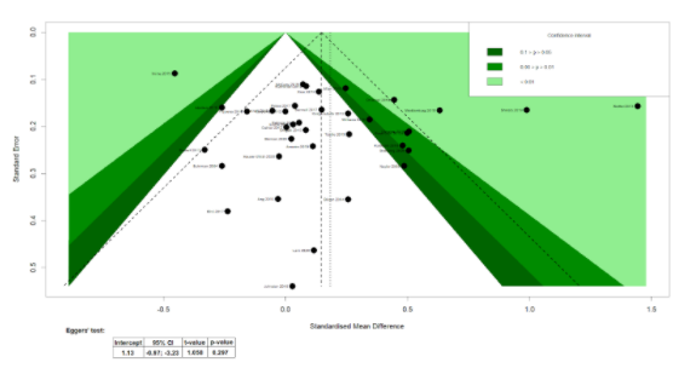


Figure S2. Funnel plot disability and function.


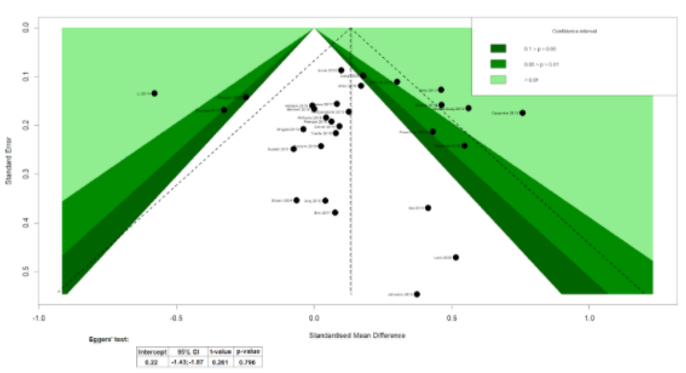


Figure S3. Funnel plot quality of life.


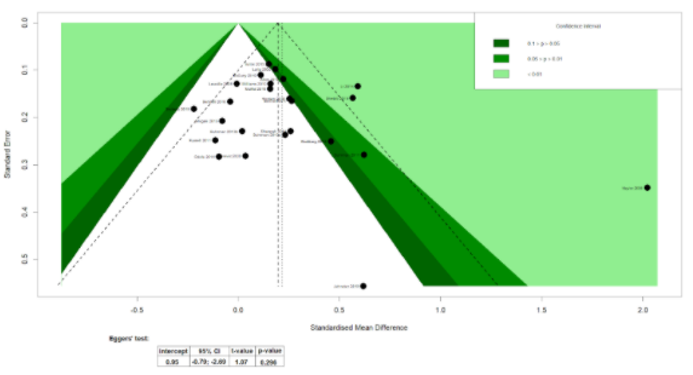


Figure S4. Funnel plot emotional functioning.


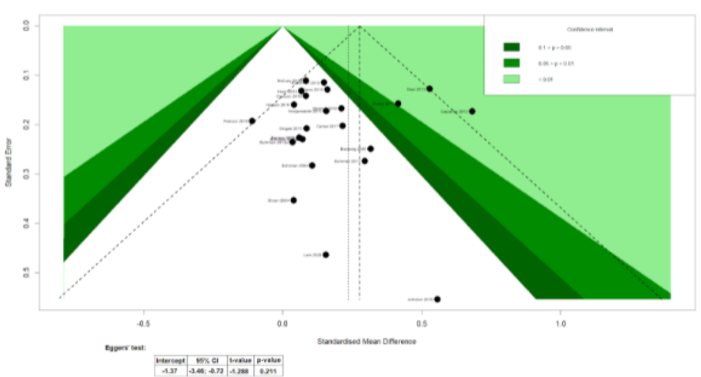


Figure S5. Funnel plot self-management.


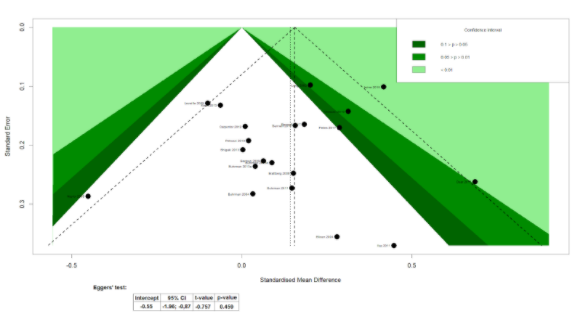


# References

1. Sebbag E, Felten R, Sagez F, Sibilia J, Devilliers H, Arnaud L. The world-wide burden of musculoskeletal diseases: a systematic analysis of the World Health Organization Burden of Diseases Database. Ann Rheum Dis 2019 Jun;78(6):844–848. PMID:30987966
2. Whiteford HA, Degenhardt L, Rehm J, Baxter AJ, Ferrari AJ, Erskine HE, Charlson FJ, Norman RE, Flaxman AD, Johns N, Burstein R, Murray CJL, Vos T. Global burden of disease attributable to mental and substance use disorders: findings from the Global Burden of Disease Study 2010. Lancet 2013 Nov 9;382(9904):1575–1586. PMID:23993280
3. Briggs AM, Towler SCB, Speerin R, March LM. Models of care for musculoskeletal health in Australia: now more than ever to drive evidence into health policy and practice. Aust Health Rev 2014 Sep;38(4):401–405. PMID:25086678
4. Dario AB, Moreti Cabral A, Almeida L, Ferreira ML, Refshauge K, Simic M, Pappas E, Ferreira PH. Effectiveness of telehealth-based interventions in the management of non-specific low back pain: a systematic review with meta-analysis. Spine J 2017 Sep;17(9):1342–1351. PMID:28412562
5. Hewitt S, Sephton R, Yeowell G. The Effectiveness of Digital Health Interventions in the Management of Musculoskeletal Conditions: Systematic Literature Review. J Med Internet Res 2020 Jun 5;22(6):e15617. PMID:32501277
6. Jiang S, Xiang J, Gao X, Guo K, Liu B. The comparison of telerehabilitation and face-to-face rehabilitation after total knee arthroplasty: A systematic review and meta-analysis. J Telemed Telecare 2018 May;24(4):257–262. PMID:28027679
7. Cottrell MA, Galea OA, O’Leary SP, Hill AJ, Russell TG. Real-time telerehabilitation for the treatment of musculoskeletal conditions is effective and comparable to standard practice: a systematic review and meta-analysis. Clin Rehabil 2017 May;31(5):625–638. PMID:27141087
8. Slattery BW, Haugh S, Francis K, O’Connor L, Barrett K, Dwyer CP, O’Higgins S, Egan J, McGuire BE. Protocol for a systematic review with network meta-analysis of the modalities used to deliver eHealth interventions for chronic pain [Internet]. Systematic Reviews. 2017. [doi: 10.1186/s13643-017-0414-x]
9. Heapy AA, Higgins DM, Cervone D, Wandner L, Fenton BT, Kerns RD. A Systematic Review of Technology-assisted Self-Management Interventions for Chronic Pain. Clin J Pain 2015;31(6):470–492.
10. Kelly M, Fullen B, Martin D, McMahon S, McVeigh JG. eHealth interventions to support self-management in people with musculoskeletal disorders: a scoping review protocol. JBI Evid Synth 2021 Mar 1;19(3):709–720. PMID:33725715
11. WHO guideline Recommendations on Digital Interventions for Health System Strengthening. Geneva: World Health Organization; 2019. ISBN:9789241550505
12. Asthana S, Jones R, Sheaff R. Why does the NHS struggle to adopt eHealth innovations? A review of macro, meso and micro factors. BMC Health Serv Res 2019 Dec 21;19(1):984. PMID:31864370
13. Valentijn PP, Schepman SM, Opheij W, Bruijnzeels MA. Understanding integrated care: a comprehensive conceptual framework based on the integrative functions of primary care. Int J Integr Care Ubiquity Press, Ltd.; 2013 Jan;13(1):e010. PMID:23687482
14. Liberati A, Altman DG, Tetzlaff J, Mulrow C, Gøtzsche PC, Ioannidis JPA, Clarke M, Devereaux PJ, Kleijnen J, Moher D. The PRISMA statement for reporting systematic reviews and meta-analyses of studies that evaluate health care interventions: explanation and elaboration. J Clin Epidemiol 2009 Oct;62(10):e1–34. PMID:19631507
15. Cumpston M, Li T, Page MJ, Chandler J, Welch VA, Higgins JPT, Thomas J. Updated guidance for trusted systematic reviews: a new edition of the Cochrane Handbook for Systematic Reviews of Interventions [Internet]. Cochrane Database of Systematic Reviews. 2019. [doi: 10.1002/14651858.ed000142]
16. Covidence. Covidence. Accelerate Your Systematic Review. 2017;
17. Dworkin RH, Turk DC, Farrar JT, Haythornthwaite JA, Jensen MP, Katz NP, Kerns RD, Stucki G, Allen RR, Bellamy N, Carr DB, Chandler J, Cowan P, Dionne R, Galer BS, Hertz S, Jadad AR, Kramer LD, Manning DC, Martin S, McCormick CG, McDermott MP, McGrath P, Quessy S, Rappaport BA, Robbins W, Robinson JP, Rothman M, Royal MA, Simon L, Stauffer JW, Stein W, Tollett J, Wernicke J, Witter J, IMMPACT. Core outcome measures for chronic pain clinical trials: IMMPACT recommendations. Pain 2005 Jan;113(1-2):9–19. PMID:15621359
18. Piot M. Statistics in Climate Sciences Chapter 11: Clustering, Distance Methods and Ordination. 2014.
19. Hair JF. Multivariate Data Analysis. 2009 [cited 2022 Feb 12]; Available from: https://digitalcommons.kennesaw.edu/facpubs/2925/
20. Valentijn PP, Ruwaard D, Vrijhoef HJM, de Bont A, Arends RY, Bruijnzeels MA. Collaboration processes and perceived effectiveness of integrated care projects in primary care: a longitudinal mixed-methods study [Internet]. BMC Health Services Research. 2015. [doi: 10.1186/s12913-015-1125-4]
21. Leznik M, Tofallis C. Estimating invariant principal components using diagonal regression. University of Hertfordshire; 2005 [cited 2022 May 11]; Available from: http://uhra.herts.ac.uk/handle/2299/715
22. Gabriel KR. The biplot graphic display of matrices with application to principal component analysis. Biometrika Oxford Academic; 1971 Dec 1;58(3):453–467.
23. Kassambara A. Practical Guide to Cluster Analysis in R: Unsupervised Machine Learning. CreateSpace Independent Publishing Platform; 2017. ISBN:9781542462709
24. Higgins JPT, Thomas J, Chandler J, Cumpston M, Li T, Page MJ, Welch VA. Cochrane Handbook for Systematic Reviews of Interventions. John Wiley & Sons; 2019. ISBN:9781119536611
25. Valentijn PP, Pereira FA, Ruospo M, Palmer SC, Hegbrant J, Sterner CW, Vrijhoef HJM, Ruwaard D, Strippoli GFM. Person-Centered Integrated Care for Chronic Kidney Disease: A Systematic Review and Meta-Analysis of Randomized Controlled Trials. Clin J Am Soc Nephrol 2018 Mar 7;13(3):375–386. PMID:29438975
26. Higgins JPT, Thompson SG, Deeks JJ, Altman DG. Measuring inconsistency in meta-analyses. BMJ British Medical Journal Publishing Group; 2003 Sep 4;327(7414):557–560. PMID:12958120
27. Egger M, Davey Smith G, Schneider M, Minder C. Bias in meta-analysis detected by a simple, graphical test. BMJ 1997 Sep 13;315(7109):629–634. PMID:9310563
28. Team RC, Others. R: A language and environment for statistical computing. Vienna, Austria; 2013; Available from: http://r.meteo.uni.wroc.pl/web/packages/dplR/vignettes/intro-dplR.pdf
29. Guyatt GH, Oxman AD, Vist GE, Kunz R, Falck-Ytter Y, Alonso-Coello P, Schünemann HJ, GRADE Working Group. GRADE: an emerging consensus on rating quality of evidence and strength of recommendations. BMJ 2008 Apr 26;336(7650):924–926. PMID:18436948
30. Slattery BW, Haugh S, O’Connor L, Francis K, Dwyer CP, O’Higgins S, Egan J, McGuire BE. An evaluation of the effectiveness of the modalities used to deliver electronic health interventions for chronic pain: systematic review with network meta-analysis. J Med Internet Res JMIR Publications Inc., Toronto, Canada; 2019;21(7):e11086.
31. Moman RN, Dvorkin J, Pollard EM, Wanderman R, Murad MH, Warner DO, Hooten WM. A systematic review and meta-analysis of unguided electronic and mobile health technologies for chronic pain—is it time to start prescribing electronic health applications? Pain Med Oxford University Press; 2019;20(11):2238–2255.
32. de Oliveira Lima L, Saragiotto BT, Costa LOP, Nogueira LC, Meziat-Filho N, Reis FJJ. Self-Guided Web-Based Pain Education for People With Musculoskeletal Pain: A Systematic Review and Meta-Analysis. Phys Ther [Internet] 2021 Oct 1;101(10). PMID:34174081
33. Paganini S, Lin J, Kählke F, Buntrock C, Leiding D, Ebert DD, Baumeister H. A guided and unguided internet- and mobile-based intervention for chronic pain: health economic evaluation alongside a randomised controlled trial [Internet]. BMJ Open. 2019. p. e023390. [doi: 10.1136/bmjopen-2018-023390]
34. Fatoye F, Gebrye T, Fatoye C, Mbada CE, Olaoye MI, Odole AC, Dada O. The Clinical and Cost-Effectiveness of Telerehabilitation for People With Nonspecific Chronic Low Back Pain: Randomized Controlled Trial. JMIR Mhealth Uhealth 2020;8(6):e15375.
35. Takeshima N, Sozu T, Tajika A, Ogawa Y, Hayasaka Y, Furukawa TA. Which is more generalizable, powerful and interpretable in meta-analyses, mean difference or standardized mean difference? BMC Med Res Methodol 2014 Feb 21;14:30. PMID:24559167
36. Deeks JJ, Higgins JPT, Altman DG. Chapter 9: analyzing data and undertaking meta-analyses. Cochrane handbook for systematic reviews of interventions version 2011;5(0).
37. Cochrane Training. Identifying publication bias in meta-analyses of continuous outcomes [Internet]. Cochrane Training. 2020 [cited 2022 Jun 24]. Available from: https://training.cochrane.org/resource/identifying-publication-bias-meta-analyses-continuous-outcomes
38. Brattberg G. Self-administered EFT (Emotional Freedom Techniques) in individuals with fibromyalgia: A randomized trial. Integr Med 2008;7(4):30–35.
39. Allen KD, Arbeeva L, Callahan LF, Golightly YM, Goode AP, Huffman KM, Severson HH, Schwartz TA, Hill C, Hill C, Hill C, Service, Rehabilitation, Hill C, Hill C. Physical therapy vs internet based exercise training for patients with knee osteoarthritis: results of a RCT. Health research alliance 2018;26(3):383–396.
40. Lorig KR, Laurent DD, Deyo RA, Marnell ME, Minor MA, Ritter PL. Can a back pain e-mail discussion group improve health status and lower health care costs? A randomized study. Arch Intern Med 2002;162(7):792–796.
41. Buhrman M, Fredriksson A, Edström G, Shafiei D, Tärnqvist C, Ljótsson B, Hursti T, Gordh T, Andersson G. Guided Internet-delivered cognitive behavioural therapy for chronic pain patients who have residual symptoms after rehabilitation treatment: Randomized controlled trial. Eur J Pain 2013;17(5):753–765.
42. Hørdam B, Sabroe S, Pedersen PU, Mejdahl S, Søballe K. Nursing intervention by telephone interviews of patients aged over 65 years after total hip replacement improves health status: A randomised clinical trial. Scand J Caring Sci 2010;24(1):94–100.
43. Buhrman M, Skoglund A, Husell J, Bergström K, Gordh T, Hursti T, Bendelin N, Furmark T, Andersson G. Guided internet-delivered acceptance and commitment therapy for chronic pain patients: A randomized controlled trial. Behav Res Ther 2013;51(6):307–315.
44. Bennell KL, Nelligan RK, Rini C, Keefe FJ, Kasza J, Frencha S, Forbes A, Dobson F, Abbott JH, Dalwood A, Harris A, Vicenzino B, Hodges PW, Hinman RS. Effects of internet-based pain coping skills training before home exercise for individuals with hip osteoarthritis (HOPE trial): A randomised controlled trial. 2018. p. 1833–1842.
45. Berman RLH, Iris MA, Bode R, Drengenberg C. The Effectiveness of an Online Mind-Body Intervention for Older Adults With Chronic Pain. Journal of Pain American Pain Society; 2009;10(1):68–79.
46. Buhrman M, Fältenhag S, Ström L, Andersson G. Controlled trial of Internet-based treatment with telephone support for chronic back pain. Pain 2004;111(3):368–377.
47. Peters ML, Smeets E, Feijge M, Van Breukelen G, Andersson G, Buhrman M, Linton SJ. Happy Despite Pain: A Randomized Controlled Trial of an 8-Week Internet-delivered Positive Psychology Intervention for Enhancing Well-being in Patients with Chronic Pain. Clin J Pain 2017;33(11):962–975.
48. Chhabra HS, Sharma S, Verma S. Smartphone app in self-management of chronic low back pain: a randomized controlled trial. Eur Spine J Springer Berlin Heidelberg; 2018;27(11):2862–2874.
49. Irvine AB, Russell H, Manocchia M, Mino DE, Glassen TC, Morgan R, Gau JM, Birney AJ, Ary DV. Mobile-web app to self-manage low back pain: Randomized controlled trial. 2015.
50. Calner T, Nordin C, Eriksson MK, Nyberg L, Gard G, Michaelson P. Effects of a self-guided, web-based activity programme for patients with persistent musculoskeletal pain in primary healthcare: A randomized controlled trial. Eur J Pain 2017;21(6):1110–1120.
51. Mecklenburg G, Smittenaar P, Erhart-Hledik JC, Perez DA, Hunter S. Effects of a 12-week digital care program for chronic knee pain on pain, mobility, and surgery risk: Randomized controlled trial. 2018.
52. Johnston M, Foster M, Shennan J, Starkey NJ, Johnson A. The effectiveness of an acceptance and commitment therapy self-help intervention for chronic pain. Clin J Pain 2010;26(5):393–402.
53. Blixen CE, Bramstedt KA, Hammel JP, Tilley BC. A pilot study of health education via a nurse-run telephone self-management programme for elderly people with osteoarthritis. J Telemed Telecare 2004;10(1):44–49.
54. Ang DC, Chakr R, Mazzuca S, France CR, Steiner J, Stump T. Cognitive-behavioral therapy attenuates nociceptive responding in patients with fibromyalgia: A pilot study. Arthritis Care Res 2010;62(5):618–623.
55. Shigaki CL, Smarr KL, Siva C, Ge B, Musser D, Johnson R. RAHelp: An online intervention for individuals with rheumatoid arthritis. Arthritis Care Res 2013;65(10):1573–1581.
56. Petrozzi MJ, Leaver A, Ferreira PH, Rubinstein SM, Jones MK, Mackey MG. Addition of MoodGYM to physical treatments for chronic low back pain: A randomized controlled trial. Chiropr Man Therap Chiropractic & Manual Therapies; 2019;27(1):1–12.
57. Shebib R, Bailey JF, Smittenaar P, Perez DA, Mecklenburg G, Hunter S. Randomized controlled trial of a 12-week digital care program in improving low back pain. npj Digital Medicine Springer US; 2019;2(1):1–8.
58. Toelle TR, Utpadel-Fischler DA, Haas K-K, Priebe JA. App-based multidisciplinary back pain treatment versus combined physiotherapy plus online education: a randomized controlled trial. npj Digital Medicine Springer US; 2019;2(1):1–9.
59. Odole AC, Ojo OD. Is telephysiotherapy an option for improved quality of life in patients with osteoarthritis of the knee? Int J Telemed Appl [Internet] 2014;2014. [doi: 10.1155/2014/903816]
60. Krein SL, Kadri R, Hughes M, Kerr EA, Piette JD, Holleman R, Kim HM, Richardson CR. Pedometer-based internet-mediated intervention for adults with chronic low back pain: Randomized controlled trial. J Med Internet Res [Internet] 2013;15(8). [doi: 10.2196/jmir.2605]
61. Chiauzzi E, Pujol LA, Wood M, Bond K, Black R, Yiu E, Zacharoff K. PainACTION-Back Pain: A self-management website for people with chronic back pain. Pain Med 2010;11(7):1044–1058.
62. Buhrman M, Nilsson-Ihrfelt E, Jannert M, Ström L, Andersson G. Guided internet-based cognitive behavioural treatment for chronic back pain reduces pain catastrophizing: A randomized controlled trial. J Rehabil Med 2011;43(6):500–505.
63. Bennell KL, Nelligan R, Dobson F, Rini C, Keefe F, Kasza J, French S, Bryant C, Dalwood A, Abbott JH, Hinman RS. Effectiveness of an internet-delivered exercise and pain-coping skills training intervention for persons with chronic knee pain: A randomized trial. Ann Intern Med 2017;166(7):453–462.
64. Leveille SG, Huang A, Tsai SB, Allen M, Weingart SN, Iezzoni LI. Health coaching via an internet portal for primary care patients with chronic conditions a randomized controlled trial. Med Care 2009;47(1):41–47.
65. Williams DA, Kuper D, Segar M, Mohan N, Sheth M, Clauw DJ. Internet-enhanced management of fibromyalgia: A randomized controlled trial. Pain 2010;151(3):694–702.
66. Allen KD, Oddone EZ, MHSc, Coffman CJ, Datta SK, Karen A. Juntilla M, Jennifer H. Lindquist M, Tessa A. Walker MPH, Weinberger M, Bosworth AHB. Telephone-Based Self-management of Osteoarthritis A Randomized Trial. Ann Intern Med 2010;153:570–579.
67. Linda S. Ruehlmana PK, Craig E. A randomized controlled evaluation of an online chronic pain self management program. Pain 2012;153(2):319–330.
68. Carpenter KM, Stoner SA, Mundt JM, Stoelbc B. An online self-help CBT intervention for chronic lower back pain. Clin J Pain 2012;28(1):14–22.
69. Dear BF, Titov N, Perry KN, Johnston L, Wootton BM, Terides MD, Rapee RM, Hudson JL. The Pain Course: A randomised controlled trial of a clinician-guided Internet-delivered cognitive behaviour therapy program for managing chronic pain and emotional well-being. Pain International Association for the Study of Pain; 2013;154(6):942–950.
70. Naylor MR, Keefe FJ, Brigidi B, Naud S, Helzer JE. Therapeutic Interactive Voice Response for chronic pain reduction and relapse prevention. Pain 2008;134(3):335–345.
71. Pozo-Cruz BD, Adsuar JC, Parraca J, Pozo-Cruz JD, Moreno A, Gusi N. A web-based intervention to improve and prevent low back pain among office workers: A randomized controlled trial. J Orthop Sports Phys Ther 2012;42(10):831–841.
72. Kristjánsdóttir ÓB, Fors EA, Eide E, Finset A, Stensrud TL, Van Dulmen S, Wigers SH, Eide H. A smartphone-based intervention with diaries and therapist feedback to reduce catastrophizing and increase functioning in women with chronic widespread pain. part 2: 11-Month follow-up results of a randomized trial. J Med Internet Res [Internet] 2013;15(3). [doi: 10.2196/jmir.2442]
73. Janevic MR, Shute V, Murphy SL, Piette JD. Acceptability and Effects of Commercially Available Activity Trackers for Chronic Pain Management Among Older African American Adults. Pain Med 2020;21(2):e68–e78.
74. McCurry SM, Von Korff M, Morin CM, Cunningham A, Pike KC, Thakral M, Wellman R, Yeung K, Zhu W, Vitiello MV. Telephone interventions for co-morbid insomnia and osteoarthritis pain: The OsteoArthritis and Therapy for Sleep (OATS) randomized trial design. Contemp Clin Trials 2019;87:105851.
75. Hauser-Ulrich S, Künzli H, Meier-Peterhans D, Kowatsch T. A Smartphone-Based Health Care Chatbot to Promote Self-Management of Chronic Pain (SELMA): Pilot Randomized Controlled Trial. JMIR Mhealth Uhealth 2020;8(4):e15806.
76. Kohns DJ, Urbanik CP, Geisser ME, Schubiner H, Lumley MA. The Effects of a Pain Psychology and Neuroscience Self-Evaluation Internet Intervention: A Randomized Controlled Trial. Clin J Pain 2020;36(9):683–692.
77. Lam J, Svensson P, Alstergren P. Internet-Based Multimodal Pain Program With Telephone Support for Adults With Chronic Temporomandibular Disorder Pain: Randomized Controlled Pilot Trial. J Med Internet Res 2020;22(10):e22326.
78. Amorim AB, Pappas E, Simic M, Ferreira ML, Jennings M, Tiedemann A, Carvalho ESAP, Caputo E, Kongsted A, Ferreira PH. Integrating Mobile-health, health coaching, and physical activity to reduce the burden of chronic low back pain trial (IMPACT): a pilot randomised controlled trial. BMC Musculoskelet Disord 2019;20(1):71.
79. Richard L. Skolasky AMMDLLHR III, Stephen TW. Health Behavior Change Counseling in Surgery for Degenerative Lumbar Spinal Stenosis. Part I: Improvement in Rehabilitation Engagement and Functional Outcomes. Arch Phys Med Rehabil 2015;96(7):1200–1207.
80. Sharareh B, Schwarzkopf R. Effectiveness of telemedical applications in postoperative follow-up after total joint arthroplasty. J Arthroplasty Elsevier Inc.; 2014;29(5):918–922.e1.
81. Moffet H, Tousignant M, Nadeau S, Mérette C, Boissy P, Corriveau H, Marquis F, Cabana F, Ranger P, Belzile ÉL, Dimentberg R. In-home telerehabilitation compared with faceto-face rehabilitation after total knee arthroplasty: A noninferiority randomized controlled trial. Journal of Bone and Joint Surgery - American Volume 2015;97(14):1129–1141.
82. Piqueras M, Marco E, Coll M, Escalada F, Ballester A, Cinca C, Belmonte R, Muniesa JM. Effectiveness of an interactive virtual telerehabilitation system in patients after total knee arthroplasty: A randomized controlled trial. J Rehabil Med 2013;45(4):392–396.
83. Bini SA, Mahajan J. Clinical outcomes of remote asynchronous telerehabilitation are equivalent to traditional therapy following total knee arthroplasty: A randomized control study. J Telemed Telecare 2017;23(2):239–247.
84. Russell TG, Buttrum P, Wootton R, Jull GA. Internet-based outpatient telerehabilitation for patients following total knee arthroplasty: A randomized controlled trial. Journal of Bone and Joint Surgery - Series A 2011;93(2):113–120.
85. Li LL, Gan YY, Zhang LN, Wang YB, Zhang F, Qi JM. The effect of post-discharge telephone intervention on rehabilitation following total hip replacement surgery. International Journal of Nursing Sciences Elsevier Ltd; 2014;1(2):207–211.
86. Tousignant M, Moffet H, Boissy P, Corriveau H, Cabana F, Marquis F. A randomized controlled trial of home telerehabilitation for post-knee arthroplasty. J Telemed Telecare 2011;17(4):195–198.
87. Iles R, Taylor NF, Davidson M, O’Halloran P. Telephone coaching can increase activity levels for people with non-chronic low back pain: A randomised trial. J Physiother Elsevier; 2011;57(4):231–238.
88. Priebe JA, Haas KK, Moreno Sanchez LF, Schoefmann K, Utpadel-Fischler DA, Stockert P, Thoma R, Schiessl C, Kerkemeyer L, Amelung V, Jedamzik S, Reichmann J, Marschall U, Toelle TR. Digital Treatment of Back Pain versus Standard of Care: The Cluster-Randomized Controlled Trial, Rise-uP. J Pain Res 2020;13:1823–1838.
89. Kosterink SM, Huis in’t Veld RMHA, Cagnie B, Hasenbring M, Vollenbroek-Hutten MMR. The clinical effectiveness of a myofeedback-based teletreatment service in patients with non-specific neck and shoulder pain: A randomized controlled trial. J Telemed Telecare 2010;16(6):316–321.
90. Pozo-cruz B, Gusi N, Pozo-cruz J, Adsuar JC, Hernandez-mocholí M, Parraca JA. Clinical effects of a nine-month web-based intervention in subacute non-specific low back pain patients : a randomized controlled trial. Clinical Rehabilitation [Internet] 2012; [doi: 10.1177/0269215512444632]
91. Pozo-cruz B, Parraca JA, Pozo-cruz J, Adsuar JC, Hill JC, Gusi N. An occupational , internet-based intervention to prevent chronicity in subacute lower back pain : a randomiZed controlled trial. 2012;581–587.
